# Production of Glycopeptide Derivatives for Exploring Substrate Specificity of Human OGA Toward Sugar Moiety

**DOI:** 10.3389/fchem.2018.00646

**Published:** 2019-01-14

**Authors:** Shanshan Li, Jiajia Wang, Lanlan Zang, Hailiang Zhu, Jianshuang Guo, Jiabin Zhang, Liuqing Wen, Yi Chen, Yanhong Li, Xi Chen, Peng George Wang, Jing Li

**Affiliations:** ^1^School of Basic Medical Sciences, Henan University Joint National Laboratory for Antibody Drug Engineering, Kaifeng, China; ^2^Department of Chemistry and Center of Diagnostics & Therapeutics, Georgia State University, Atlanta, GA, United States; ^3^Central Laboratory, Linyi People's Hospital, Shandong University, Linyi, China; ^4^State Key Laboratory of Medicinal Chemical Biology, College of Pharmacy and Tianjin Key Laboratory of Molecular Drug Research, Nankai University, Tianjin, China; ^5^Department of Chemistry, University of California, Davis, Davis, CA, United States

**Keywords:** *O*-GlcNAcylation, *O*-GlcNAcase, sugar moiety, GlcNAc derivatives, substrate specificity

## Abstract

*O*-GlcNAcase (OGA) is the only enzyme responsible for removing *N*-acetyl glucosamine (GlcNAc) attached to serine and threonine residues on proteins. This enzyme plays a key role in *O*-GlcNAc metabolism. However, the structural features of the sugar moiety recognized by human OGA (hOGA) remain unclear. In this study, a set of glycopeptides with modifications on the GlcNAc residue, were prepared in a recombinant full-length human OGT-catalyzed reaction, using chemoenzymatically synthesized UDP-GlcNAc derivatives. The resulting glycopeptides were used to evaluate the substrate specificity of hOGA toward the sugar moiety. This study will provide insights into the exploration of probes for *O*-GlcNAc modification, as well as a better understanding of the roles of O-GlcNAc in cellular physiology.

## Introduction

Protein glycosylation is an important protein post-translational modification in various organisms and is critical for a wide range of biological processes. Defective or aberrant glycosylation can lead to serious cellular dysfunction or diseases in humans (Wells et al., 2001). *O*-GlcNAcylation is a single *N*-acetylglucosamine beta-linked to the serine or threonine residues of some nucleic acids and cytoplasmic proteins, and is essential for multiple cellular signaling cascades, including gene transcription, protein translation, cell cycle, and nutrient sensing (Chen et al., 2013; Ferrer et al., 2014; Hardiville and Hart, 2014). By affecting the stability, activity, or interactions of key signaling proteins, *O*-GlcNAcylation is a novel and vital factor in the cause of diseases such as diabetes, cancer, and neurodegeneration (Yuzwa et al., 2012; Yuzwa and Vocadlo, 2014; Zhu et al., 2014). Because of the importance of *O*-GlcNAc and its crucial role in disease pathology, the enzymes controlling *O*-GlcNAcylation levels are considered to be therapeutic targets.

*O*-GlcNAcylation is a dynamic reversible process, the addition and removal of GlcNAc residues from a protein is regulated by only two enzymes. Uridine diphosphate-*N*-acetyl-D-glucosamine: polypeptidyl transferase (OGT) is responsible for transferring a single GlcNAc from uridine diphosphate-α-*N*-acetyl-D-glucosamine (UDP-GlcNAc) to proteins. *O*-GlcNAcase (OGA) removes GlcNAc from glycoproteins. Substrate promiscuity of OGT and OGA has been shown to be valuable for enzymatic catalysis and *O*-GlcNAcylation level control. The substrate specificity of human OGT (hOGT) has been widely investigated and the hOGT crystal structure has been solved (Lazarus et al., 2011). Among the 26 UDP-sugar derivatives, 4 (UDP-GlcNPr, UDP-6-deoxy-GlcNAc, UDP-4-deoxy-GlcNAc and UDP-6-deoxy-GalNAc) were reported to highly glycosylate peptides via sOGT-catalyzed glycosylation (Ma et al., 2013). For peptide acceptor recognition, 3 positions (−2, −1, and +2) in the peptide sequence are vital for *O*-GlcNAcylation, and uncharged amino acids are preferred and show high reactivity (Liu et al., 2014). Recently, several research groups solved the structure of hOGA and revealed that hOGA existed as an unusual arm-in-arm homodimer. The residues of hOGA on the cleft surface enabled broad interactions with peptide substrates, indicating the important role of the peptide structure in the recognition mode (Elsen et al., 2017; Li et al., 2017; Roth et al., 2017). However, partly because of the difficulties in obtaining glycopeptides with modified GlcNAc residues, structure determination during GlcNAc-recognition by hOGA remains unclear.

We previously evaluated chemoenzymatic synthesis and purified oligosaccharides and glycopeptides (Wen et al., 2016a,b; Wu et al., 2016; Wang et al., 2018). As an extension of these studies, glycopeptides containing different GlcNAc derivatives were successfully synthesized and purified. Further, we investigated the substrate specificity of hOGA and found that hOGA was less promiscuous in tolerating substrates with modified GlcNAc.

## Results

### Synthesis of GlcNAc Derivatives

**Figure 1:**
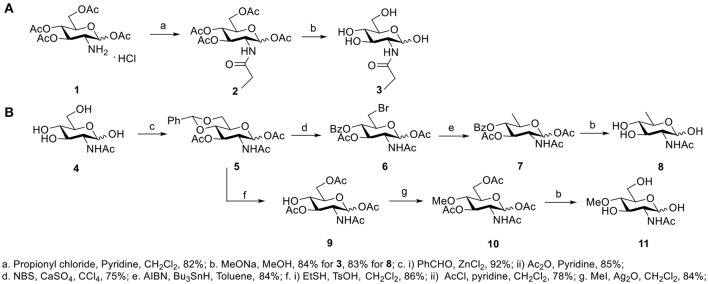
Synthetic methods of compounds: **(A)** GlcNPr, **(B)** 4-OMe-GlcNAc, and 6-deoxy-GlcNAc.

GlcNAc and GalNAc were purchased from *Carbosynth* (San Diego, CA, USA). GlcNPh, GlcNGc, GlcNTFA, and 6-N_3_-GlcNAc were gifted from Dr. Xi Chen's lab (UC Davis, USA). 4-Deoxy-GlcNAc and GlcNAz were synthesized according to our previous report (Li et al., 2016). The other three new compounds, GlcNPr, 4-OMe-GlcNAc, and 6-deoxy-GlcNAc, were synthesized for the first time in this study (Figure 1). GlcNPr was generated in the presence of propionyl chloride and pyridine after global deprotection. A convergent route was designed to synthesize 6-deo-GlcNAc and 4-OMe-GlcNAc; GlcNAc as the starting material was first protected with benzylidine and then subjected to 1,3-diacetylation to afford intermediate **5**. Selective deprotection of benzylidine was carried out in the presence of NBS and calcium sulfate to afford **6**. The desired target compound **8** was obtained after reduction of the bromide and full deacetylation. For 4-OMe-GlcNAc, bezylidine in **5** was removed and selective acetylation of the 6-OH freed the 4-OH, which was *O*-methylated in the presence of iodomethane and silver oxide. **11** was obtained after global deprotection.

### Protein Expression and Purification

According to previous studies, we overexpressed *Bifidobacterium infantis N*-acetylhexosamine 1-kinase ATCC#15697 (BiNahK), human UDP-*N*-acetylgalactosamine pyrophosphorylase (hAGX1), human OGT (ncOGT), and human OGA (hOGA) in *Escherichia coli* (Gross et al., 2005; Guan et al., 2010; Li et al., 2011, 2012). Enzymes were expressed by induction with 1 mM of isopropyl 1-thio-β-D-galactopyranoside followed by incubation at 16°C for 24 h with vigorous shaking (200 rpm). After purification, 15 mg ncOGT, 20 mg hOGA, 40 mg BiNahK, and 20 mg hAGX1 were obtained from 1 L of an *E. coli* culture. As shown in Figure S1, enzymes with an N-terminal His_6_ tag showed 80% purity with a high yield. The apparent molecular weights of the enzymes according to SDS-PAGE were well-correlated with the calculated molecular weights of each protein.

### Production of Glycopeptides and Determination of Substrate Specificity of OGT

**Figure 2:**
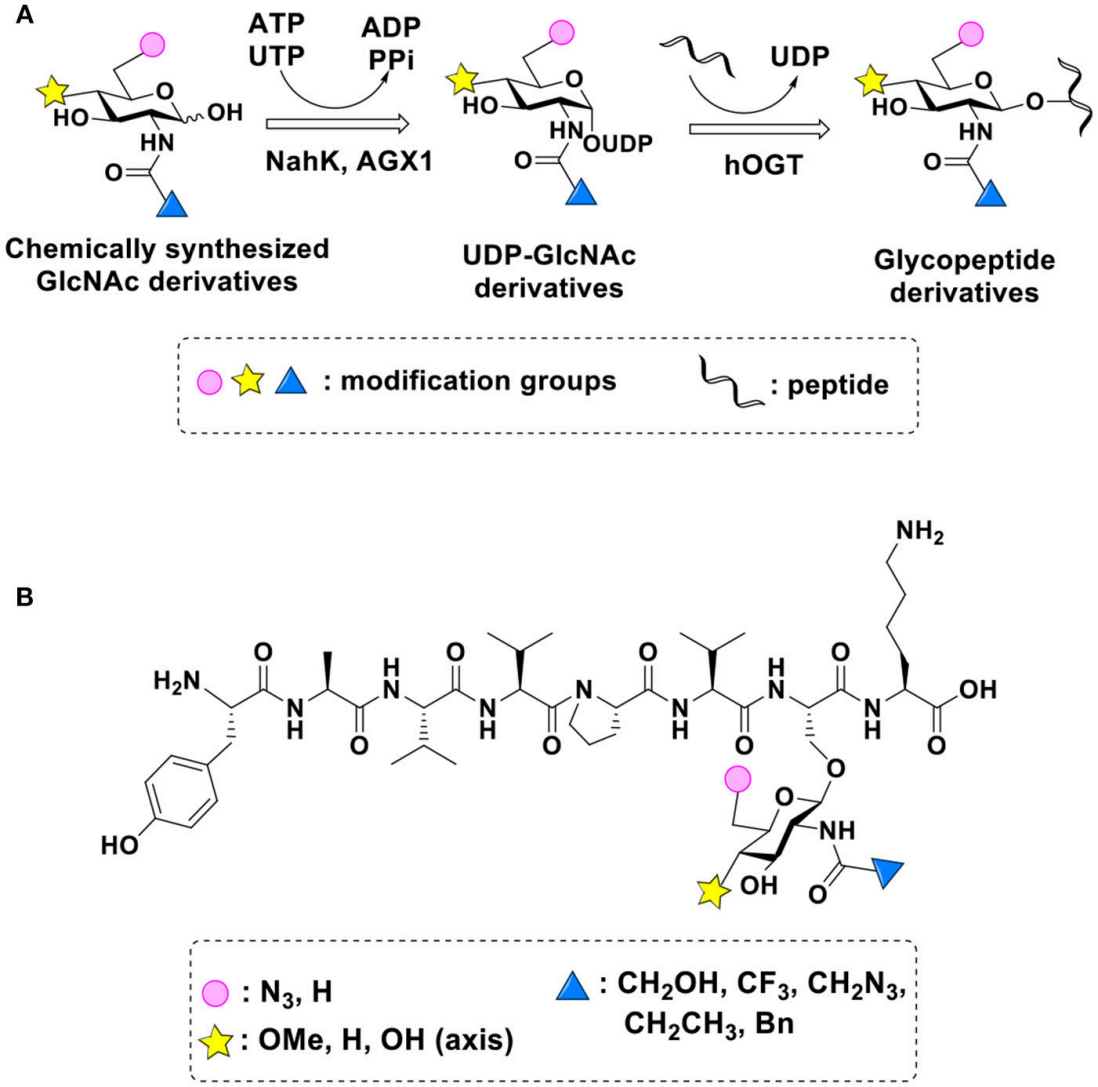
**(A)** Production of glycopeptides by hOGT-catalyzed reaction using chemoenzymatically synthesized UDP-GlcNAc derivatives. **(B)** The structures of glycopeptide derivatives used in the study.

UDP-GlcNPh, UDP-GlcNGc, UDP-GlcNTFA, and UDP-6-N_3_-GlcNAc were synthesized as reported previously (Chen et al., 2011). Other sugar nucleotides were obtained through a chemoenzymatic strategy by chemical synthesis of GlcNAc derivatives, which were used as the substrates in a one-pot two-enzyme system containing BiNahK and hAGX1 (Figure 2A). All UDP-sugars produced were characterized by capillary electrophoresis and matrix-assisted laser desorption/ionization (MALDI)-time-of-flight mass spectrometry (Figure S3).

**Figure 3:**
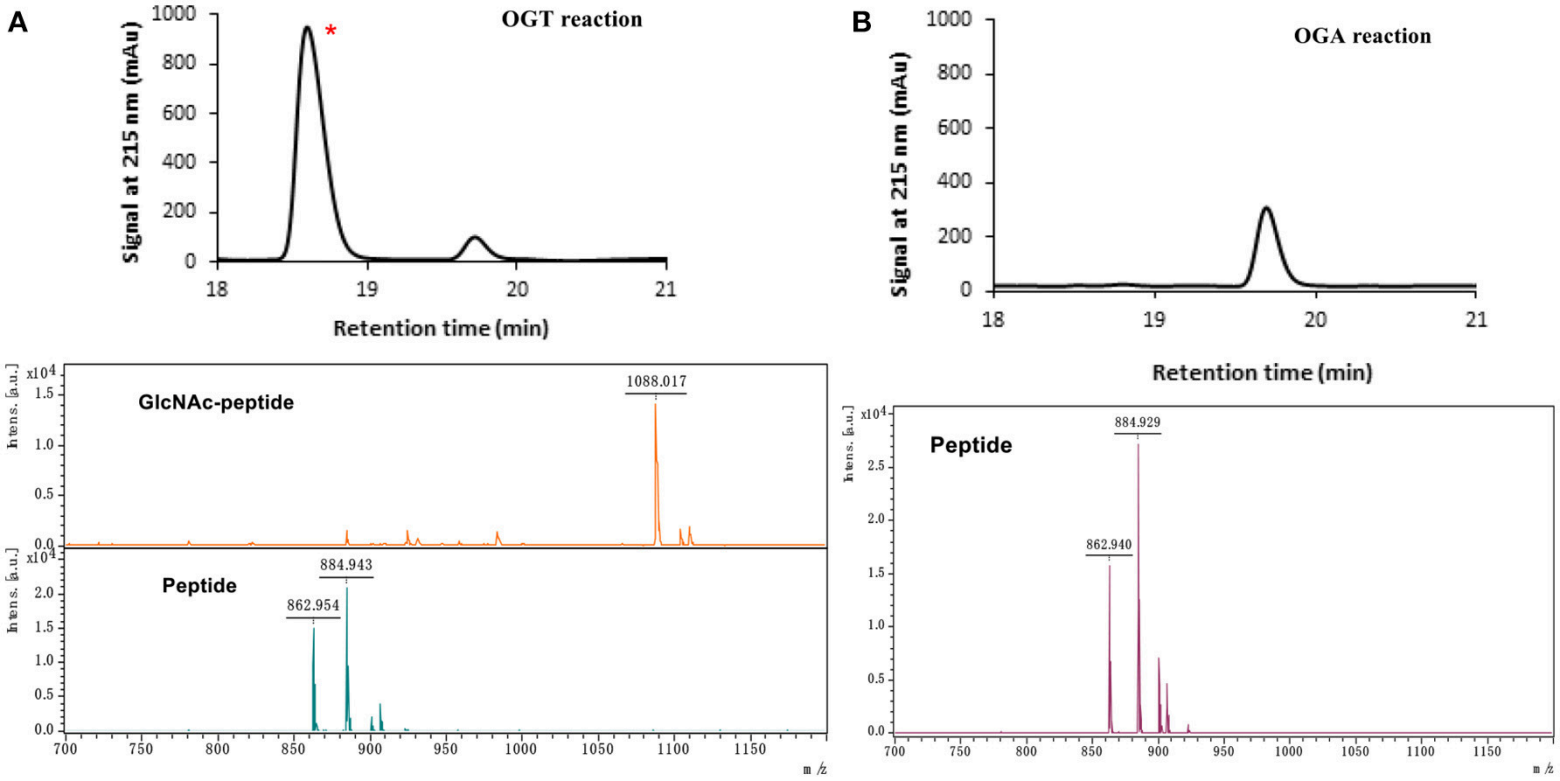
The HPLC and MALDI-TOF profile for OGT **(A)** and OGA **(B)** reactions toward UDP-GlcNAc substrates. ^*^ represents the glycopeptide peak.

The peptide substrate (YAVVPVSK) was synthesized based on the Fmoc-strategy using a Liberty Blue Peptide Synthesizer and purified by high-performance liquid chromatography (HPLC) (Liu et al., 2014) (Figure S4). A set of glycopeptides carrying modifications on the GlcNAc residue were subsequently prepared in a recombinant full-length hOGT-catalyzed reaction using chemoenzymatically synthesized UDP-GlcNAc derivatives (Figure 2A). The hOGT enzyme reactions were carried out at 37°C for 2 h in a volume of 100 μL containing 1 mM peptide, 3 mM UDP-GlcNAc analogs, and 50 μg ncOGT in buffer (50 mM Tris-HCl, pH 7.5, 10 mM Mg^2+^). The reaction was boiled, and the products were subjected to HPLC purification and yield quantification (Table 1). Each peak was collected and identified based on MALDI. For example, GlcNAc showed a peak at 18.6 min and was characterized as GlcNAc-peptide with m/z = 1088.017, while the peak at 19.7 min was characterized as a peptide with m/z = 862.954 (M+H) and 884.943 (M+Na) (Figure 3A). The retention time of the pure peptide was 19.7 min, while the GlcNAc-peptide showed a retention time of 18.6 min because of the increased polarity of GlcNAc (Figure 3A). Moreover, the OGT reaction efficiency was calculated as the glycopeptide yield (Y_a_%). The yield was calculated with the formula Y_a_ = S1/(S1+S2), where S1 and S2 are the integrated areas of the glycopeptide and peptide peaks, respectively (Table 1). The HPLC profile of the OGT and OGA reaction on the peptide are detailed in the Supporting Materials (Figure S2).

**Table 1:** The yields of OGT and OGA reactions.

**UDP-GicNAc derivatives**	**Glycopeptid yield(%)**	**OGA hydrolysis yield(%)**	**UDP-GicNAc derivatives**	**Glycopeptide yield (%)**	**OGA hydrolysis yield (%)**
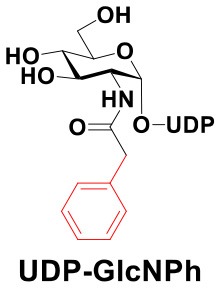	78.5 ± 1.2	ND^b^	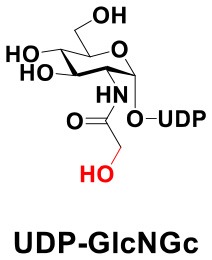	56.0 ± 0.9	5.4 ± 0.3
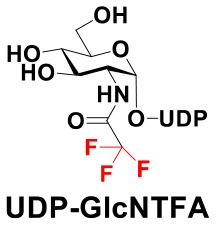	81.2 ± 1.1	3.0 ± 0.8	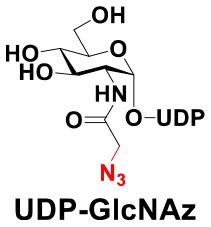	92.6 ± 3.2	96.3 ± 1.6
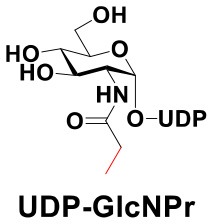	49.1 ± 2.5	ND^b^	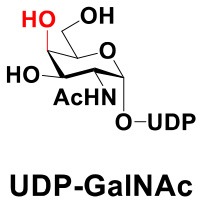	58.9 ± 0.4	47 ± 0.6
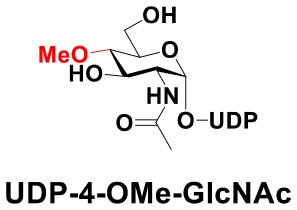	58.5 ± 1.1	ND^b^	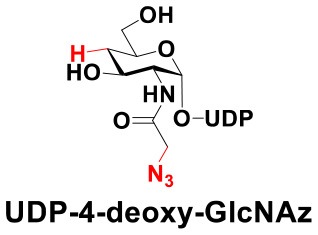	99.4 ± 0.8	ND^b^
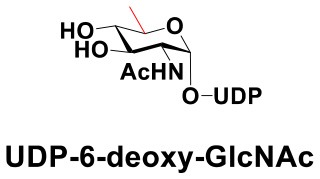	63.1 ± 0.9	ND^b^	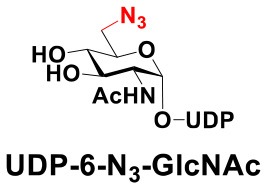	23.8 ± 1.2	62.2 ± 2.4

ND^b^: not detected

By utilizing this method, 10 glycopeptide derivatives (5 glycopeptides with C2-modified GlcNAc, 3 glycopeptides with C4-modified GlcNAc, and 2 glycopeptides with C6-modified GlcNAc) were produced (Figure 2B), purified by HPLC, and characterized by MALDI (Figure S5). All UDP-GlcNAc derivatives were good substrates for hOGT except for UDP-6-N_3_-GlcNAc, which resulted in only 23.8% yield *in vitro* (Table 1). Notably, hOGT was the full-length isoform with 13.5 tetratricopeptide repeats (TPRs), which showed higher activity than sOGT (short OGT with 2.5 TPRs). For example, when sOGT was used, six UDP-GlcNAc derivatives (UDP-GlcNGc, UDP-GlcNAz, UDP-GlcNTFA, UDP-GlcNPh, UDP-6-N_3_-GlcNAc, and UDP-GalNAc) used as the substrates produced <1% yield (Ma et al., 2013). In comparison, over 49% yield was obtained when full-length hOGT was used. The crystal structures of the N-terminal domain of full-length hOGT containing the first 11.5 TPRs, showed that the conserved asparagine residues in the TPR domain directly contacted the substrates, supporting the role of the TRP domains in initial recognition and substrate specificity determination (Jinek et al., 2004). In summary, in addition to preparing glycopeptides, we found that full-length hOGT is a more efficient catalyst than the previously reported short sOGT.

### OGA Substrate Specificity Assay

The glycopeptide peaks from OGT reactions were collected and lyophilized for the hOGA substrate specificity assay. The hOGA reaction was conducted overnight in a volume of 100 μL containing the glycopeptides, MgCl_2_ (20 mM), Tris–HCl (200 mM), and recombinant hOGA (0.1 mg). The reaction mixture was boiled and centrifuged before HPLC separation. Each peak was collected and characterized by MALDI. When the GlcNAc-peptide was treated with hOGA, efficient hydrolysis activity of hOGA and a single peptide peak were observed (Figure 3B). The hOGA hydrolysis yield (Y_b_%) was calculated using the formula Y_b_ = S2/(S1+S2), where S1 and S2 are the integrated areas of the glycopeptide and peptide peaks, respectively. The HPLC profiles of the OGT and OGA reaction on the peptide are described in detail in the Supporting Materials (Figure S2). The GlcNAz-peptide and 6-N_3_-GlcNAc-peptide were well-tolerated by hOGA, and other glycopeptide derivatives significantly decreased the efficiency of hOGA, showing <10% hydrolysis yield (Table 1). The results revealed that hOGA had a relatively strict recognition for the sugar moiety in the glycopeptides, thus exhibiting limited activities for glycopeptide derivatives with C2, C4, and C6 modifications on GlcNAc.

## Conclusion

Glycopeptides containing different GlcNAc derivatives were successfully synthesized in a recombinant full-length hOGT-catalyzed reaction, using chemoenzymatically synthesized UDP-GlcNAc derivatives. We also found that full-length hOGT is a more efficient catalyst than sOGT, as over a 49-fold yield was observed when hOGT was used. The resulting glycopeptides were used to investigate the substrate specificity of hOGA toward the sugar moiety. hOGA was less promiscuous in tolerating glycopeptide substrates with modified GlcNAc, and changes in the C2, C4, and C6 positions of GlcNAc partially affected its recognition. Interestingly, OGT and OGA possessed different levels of tolerance to the same sugar moiety, such as GlcNPh, GlcNTFA, and 4-OMe-GlcNAc. Therefore, the acquired GlcNAc derivatives, which were well-recognized by OGT but not hydrolyzed by OGA, may increase the *O*-GlcNAcylation level *in vivo* when the peracetylated compound is metabolically incorporated. Indeed, our group developed an OGA-resistant probe, peracetylated 4-deoxy-*N*-azidoacetylglucosamine (Ac_3_-4-deoxy-GlcNAz), which can be used as a potent tool for *O*-GlcNAcylation detection and the identification of *O*-GlcNAcylated proteins (Li et al., 2016). Thus, OGA-resistant GlcNAc substrates show potential as excellent probes for investigating *O*-GlcNAcylation and elucidating their importance in cellular events.

## Author Contributions

SL and JW conducted the experiments and wrote the manuscript. LZ, JG, HZ, JZ, YC, YL, and XC conducted the experiments and the sample analysis. XC and LW contributed to the manuscript revision. PW and JL designed and managed the study.

### Conflict of Interest Statement

The authors declare that the research was conducted in the absence of any commercial or financial relationships that could be construed as a potential conflict of interest.
