# Supplementary material for: Production of Glycopeptide Derivatives for Exploring Substrate Specificity of Human OGA Toward Sugar Moiety
Source: Front Chem. 2019 Jan 14;6:646. doi: 10.3389/fchem.2018.00646 (PMC6340312; doi:10.3389/fchem.2018.00646)

**Supporting Information**

**Production of glycopeptide derivatives for exploring substrate specificity of human OGA towards sugar moiety**

Shanshan Li,^b^^†^ Jiajia Wang,^a,b†^* Lanlan Zang,^d†^ Hailiang Zhu,^b^ Jianshuang Guo,^c^ Jiabin Zhang,^b^ Liuqing Wen,^b^ Yi Chen,^e^ Yanhong Li,^e^ Xi Chen,^e^ Peng George Wang,^b,c^* Jing Li^c^*

a. School of Basic Medical Sciences, Henan University Joint National Laboratory for Antibody Drug Engineering, Kaifeng, Henan, 475004, People’s Republic of China

b. Department of Chemistry and Center of Diagnostics & Therapeutics, Georgia State University, 50 Decatur St SE, Atlanta, Georgia, 30303, United States

c. State Key Laboratory of Medicinal Chemical Biology, College of Pharmacy and Tianjin Key Laboratory of Molecular Drug Research, Nankai University, Haihe Education Park, 38 Tongyan Road, Tianjin 300353, People’s Republic of China

d. Central Laboratory, Linyi People’s Hospital, Shandong University, Linyi, Shandong, 276003, People’s Republic of China

e. Department of Chemistry, University of California, One Shields Avenue, Davis, CA 95616, USA.

To whom correspondence should be addressed: Jiajia Wang, Email: jwang77@vip.henu.edu.cn; Peng George Wang, Email: [pwang11@gsu.edu](mailto:pwang11@gsu.edu); Jing Li, Email: [jinglink@nankai.edu.cn](mailto:jinglink@nankai.edu.cn);

^†^ These authors have contributed equally to this work.

Key words: *O*-GlcNAcylation, *O*-GlcNAcase, sugar moiety, GlcNAc derivatives, substrate specificity

**1. Compound synthesis**

**1.1 Synthesis of GlcNPr**

1,3,4,6-Tetraactyl Glucosamine hydrochloride (200 mg, 0.52 mmol) was dissolved in 10 mL anhydrous CH_2_Cl_2_, pyridine (82 μL, 1.04 mmol) was added at 0℃, 10 mins later, propionyl chloride (90 μL, 1.04 mmol) was added dropwise and the mixture was allowed to warm to room temperature gradually and continuously reacted at this temperature overnight. Added 1 mL MeOH to quench the reaction and the mixture was diluted with 30 mL CH_2_Cl_2_, washed with 10 mL 1M HCl and saturated sodium bicarbonate in sequence, the combined the organic layer was dried over anhydrous Na_2_SO_4_. The solvent was dried by vacuum to furnish the crude product which was purified by flashing silica gel column chromatography. The desired product was obtained as white solid **2** (172 mg, 82%). ^1^H NMR (400 MHz, CDCl_3_) δ5.82 (dd, *J* = 13.6, 9.9 Hz, 1H), 5.69 (d, *J* = 8.8 Hz, 1H), 5.19 - 5.08 (m, 2H), 4.34 – 4.22 (m, 2H), 4.11 (dd, *J* = 12.5, 2.0 Hz, 1H), 3.81 (ddd, *J* = 9.5, 4.6, 2.2 Hz, 1H), 2.15 – 2.06 (m, 8H), 2.02 – 2.01 (m, 6H), 1.07 (dd, *J* = 8.0, 7.2 Hz, 3H); ^13^C NMR (100 MHz, CDCl_3_) δ 173.97, 171.25, 170.70, 169.56, 169.30, 92.64, 72.91, 72.65, 67.87, 61.75, 52.75, 29.80, 20.85, 20.71, 20.63, 20.57, 9.85.

The intermediate **2** (172 mg) was dissolved in 10 mL dry MeOH and catalytic MeONa was added to adjust the Ph 9 ~10, the reaction was stirred at room temperature for 5h. After that, Dowex acid resin was added to make the mixture neutral. Filtrated to remove the resin and collect the filtration to afford the residue. After flashing the silica gel column to afford the desired product as colorless oil **3** (84.3 mg, 84%). ^1^H NMR (400 MHz, CD_3_OD) δ 5.03 (d, *J* = 3.4 Hz, 1H), 3.79 – 3.71 (m, 3H), 3.66 – 3.61 (m, 2H), 3.35 – 3.27 (m, 1H), 2.20 (q, *J* = 15.2, 7.7 Hz, 2H), 1.06 (t, *J* = 7.6 Hz, 3H); ^13^C NMR (100 MHz, CD_3_OD) δ 177.43, 92.57, 73.08, 72.62, 72.50, 62.81, 55.78, 30.07, 10.40.

**1.2 Synthesis of 6-deoxy GlcNAc**

Compound **5**

*N*-Acetyl-Glucosamine (8 g, 36.1 mmol) and ZnCl_2_ (5 g, 36.6 mmol) were dissolved in 20 mL dry PhCHO under the protection of N_2_, the mixture was stirred overnight at room temperature. The white solid was washed with water (2×100 mL) and hexane (2×100 mL) to afford the pure product as white solid (10.2 g, 92%).

The intermediate (10 g, 32.3 mmol) was dissolved in 100 mL dry pyridine at 0℃, and Acetyl anhydride (9.2 mL, 97 mmol) was added dropwise. The mixture was allowed to warm to room temperature and stirred overnight at this temperature. Removal of the solvent by vacuum pump and then the residue was diluted with CH_2_Cl_2_ 300 mL, the organic layer was washed with 1M HCl (100 mL), saturated NaHCO_3_ (100 mL) and brine (100 mL) sequentially, the organic layer was combined and dried over anhydrous Na_2_SO_4_. The solvent was removed to provide the crude product which was purified by flashing silica gel column chromatography to yield the pure product as white solid **5** (10.8 g, 85%)

Compound **6**

To the solution of **5** (600 mg, 1.52 mmol) in dry CCl_4_, *N*-Bromosuccinimide (407 mg, 2.29 mmol) and Calcium carbonate (167 mg, 1.67 mmol) were added. The mixture was refluxed for 4h and then cooled to the room temperature. The mixture was diluted with DCM 100 mL and washed with 1M HCl (30 mL), saturated NaHCO_3_ (30 mL) and brine (30 mL) sequentially. The combined organic layer was dried over anhydrous Na_2_SO_4_ and the solvent was removed under reduced pressure. The crude product was purified by flash chromatography, giving a colorless oil **6** (538 mg, 75%)**.** ^1^H NMR (400 MHz, CDCl_3_) δ 8.00 (dd, *J* = 8.3, 1.2 Hz, 1H), 7.63 – 7.59 (m, 1H), 7.48 – 7.44 (m, 1H), 6.24 (d, *J* = 3.7 Hz, 1H), 5.64 (d, *J* = 8.3 Hz, 1H), 5.44 – 5.36 (m, 2H), 4.56 (ddd, *J* = 10.8, 9.1, 3.7 Hz, 1H), 4.14 (ddd, *J* = 9.0, 6.0, 2.6 Hz, 1H), 3.52 – 3.39 (m, 2H), 2.23 (s, 1H), 1.95 (s, 1H), 1.94 (s, 1H); ^13^C NMR (100 MHz, CDCl_3_) δ 171.49, 169.71, 168.32, 164.67, 133.62, 129.60, 128.44, 90.32, 71.00, 70.39, 70.16, 50.85, 30.48, 22.81, 20.70, 20.39.

Compound **7**

Compound **6** (250 mg, 0.53 mmol) and azodiisobutyronitrile (AIBN, 43.5 mg, 0.26 mmol) were dissolved in dry Toluene. Bu_3_SnH (0.2 mL, 0.79 mmol) was added to the solution and the mixture was heated to 80℃ with argon protection. The reaction mixture was stirred for 5h at this temperature. TLC analysis indicated that the starting material was consumed completely. The solvent was evaporated under reduced pressure and the residue was purified using flash chromatography to provide the desired product as white solid **7** (175 mg, 84%). ^1^H NMR (400 MHz, CDCl_3_) δ 7.99 – 7.96 (m, 2H), 7.57 (t, *J* = 7.4 Hz, 1H), 7.446 – 7.41 (m, 2H), 6.14 (d, *J* = 3.7 Hz, 1H), 5.72 (d, *J* = 8.9 Hz, 1H), 5.38 – 5.32 (m, 1H), 5.15 (t, *J* = 9.7 Hz, 1H), 4.50 (ddd, *J* = 11.1, 9.1, 3.7 Hz, 1H), 4.05 – 3.98 (m, 1H), 2.19 (s, 3H), 1.92 (s, 3H), 1.90 (s, 3H), 1.22 (d, *J* = 6.4 Hz, 3H); ^13^C NMR (100 MHz, CDCl_3_) δ 171.84, 170.06, 169.01, 165.23, 133.63, 129.77, 128.63, 90.86, 73.30, 70.64, 68.15, 51.45, 23.08, 21.05, 20.70, 17.53.

Compound **8**

Compound **7**(100 mg, 0.25 mmol) was dissolved in dry Methanol and catalytic amount MeONa was added to adjust the pH 9~10. The reaction mixture was stirred at room temperature for 4h. Dowex acid resin was added to neutralize the mixture until the pH to 6~7. The solvent was evaporated in vacuo and the crude product was purified by flash silica gel chromatography to provide the pure product as colorless oil **8** (43.3 mg, 83%). ^1^H NMR (400 MHz, CD_3_OD) δ 4.97 (d, *J* = 3.5 Hz, 1H), 4.51 (d, *J* = 8.4 Hz, 0.74H), 3.83 – 3.77 (m, 1.79 H), 3.62 – 3.53 (m, 1.76 H), 3.37 – 3.34 (m, 0.75 H), 2.98 (td, *J* = 9.1, 4.0 Hz, 1.54 H), 1.94 (s, 4.64 H), 1.23 (d, *J* = 6.2 Hz, 2.1 H), 1.17 (d, *J* = 6.3 Hz, 3H); ^13^C NMR (100 MHz, CD_3_OD) δ 174.30, 173.72, 96.76, 92.41, 78.15, 77.47, 75.72, 73.23, 72.47, 68.25, 58.86, 56.11, 22.95, 22.68, 18.23, 18.18.

**1.4 Synthesis of 4-OMe-GlcNAc**

Compound **9**

The intermediate **5** (1.0 g, 2.5 mmol) and p-toluenesulfonic acid (43 mg, 0.25 mmol) were dissolved in dry DCM 50 mL, and ethanethiol (0.73 mL, 10.1 mmol) was added to the solution at room temperature. The reaction mixture was stirred at this temperature for 4h. The solvent was evaporated under reduced pressure. The crude product was purified with flash chromatography to furnish the desired product as white solid (572 mg. 75%). The whole intermediate (572 mg, 1.9 mmol) and pyridine (2.3 mL, 2.8 mmol) were suspended in dry DCM 20 mL at ice-bath, acetyl chloride (0.15 mL, 2.1 mmol) was added dropwise in 10 min. After that the ice-bath was removed to allow the mixture warm to room temperature. The mixture continually reacted for another 6h. Methanol (0.5 mL) was added to quench the excess acetyl chloride and the solvents were evaporated under reduced pressure. The crude product was purified using flash chromatography to provide the desired product as white solid **9** (568 mg, 86%). ^1^H NMR (400 MHz, CDCl_3_) δ 6.14 (d, J = 3.6 Hz, 1H), 5.91 (d, J = 8.9 Hz, 1H), 5.09 (dd, J = 10.9, 9.4 Hz, 1H), 4.51 (dd, J = 12.4, 3.6 Hz, 1H), 4.32 (ddd, J = 11.1, 9.0, 3.6 Hz, 1H), 4.20 (dd, J = 12.3, 1.9 Hz, 1H), 3.84 (d, J = 9.7 Hz, 1H), 3.66 – 3.60 (m, 1H), 3.54 (d, J = 5.4 Hz, 1H), 2.16 (s, 3H), 2.11 (s, 3H), 2.10 (s, 2H), 1.91 (s, 3H); ^13^C NMR (100 MHz, CDCl_3_) δ 172.18, 171.94, 170.65, 169.22, 90.91, 72.65, 72.17, 67.84, 62.61, 51.24, 23.01, 21.01, 20.97, 20.86.

Compound **10**

To the solution of **9** (200 mg, 0.57 mmol) and silver oxide (1.32 g, 5.7 mmol) were dissolved in dry DCM 20 mL, the flask was protected with aluminized paper, and methyl iodide (0.36 mL, 5.7 mmol) was added to the solution at ice-bath. The ice-bath was removed and gradually warm to room temperature and reacted at ambient temperature overnight. The reaction mixture was filtrated and the filtration was collected and evaporated under reduced pressure. The crude product was purified using flash chromatography to afford the pure product as white solid **10** (161 mg, 78%). 1H NMR (400 MHz, CDCl_3_) δ 6.07 (d, J = 3.6 Hz, 1H), 5.73 (d, J = 9.1 Hz, 1H), 5.16 (dd, J = 10.9, 9.2 Hz, 1H), 4.37 – 4.31 (m, 1H), 4.26 – 4.24 (m, 1H), 3.86 – 3.75 (m, 1H), 3.44 (s, 3H), 2.13 (s, 2H), 2.09 (s, 3H), 2.08 (s, 3H), 1.90 (s, 4H); ^13^C NMR (100 MHz, CDCl_3_) δ 171.65, 170.66, 170.18, 168.89, 90.84, 76.96, 73.22, 70.90, 62.35, 60.80, 51.26, 23.03, 21.01, 20.95, 20.80.

Compound **11**

Compound **10** (161 mg, 0.44 mmol) was dissolved in dry Methanol and catalytic amount MeONa was used to adjust the pH value to 9~10. The reaction mixture was stirred at room temperature overnight. Dowex acid resin was added to neutralize the mixture until the pH to 6~7. The solvent was evaporated in vacuo and the crude product was purified by flash silica gel chromatography to provide the pure product as colorless oil **11** (88 mg, 84%). ^1^H NMR (400 MHz, D_2_O) δ 5.19 (d, J = 3.2 Hz, 1H), 3.89 – 3.81 (m, 5H), 3.59 (s, 3H), 3.57 – 3.55 (m, 1H), 3.29 (t, J = 9.0 Hz, 1H), 2.06 (s, 4H); ^13^C NMR (100 MHz, D_2_O) δ 174.49, 90.69, 79.76, 70.60, 70.43, 60.34, 60.17, 54.14, 21.88.

**1.5 Synthesis of 4-Deoxy-GlcNAz and GlcNAz**

The synthesis of 4-Deoxy-GlcNAz and GlcNAz were described as previously published paper.^1^

**2. General methods**

***2.1 Synthesis of sugar nucleotides with BiNahK, and hAGX1*** The synthesis of sugar nucleotides were described as previously published paper.^(26)^ In brief, the reactions were carried out in the system containing a monosaccharide (30 mM), UTP (30 mM), ATP (30 mM), MgCl_2_ (20 mM) and Tris–HCl (200 mM), pH 8.0, recombinat NahK (0.5 mg), recombinant AGX1 (0.5 mg). After incubation at 37°C for 24 h, UDP sugar products were purified with Bio-Gel P-2 column, and further characterized by mass spectrometry and capillary electrophoresis (Fig. S2). Finally, the UDP-GlcNAc derivatives were obtained with reasonable yield (Table S1).

***2.2 Synthesis of peptide*** Automated peptide synthesis was performed on Liberty Blue Peptide Synthesizer through Fmoc-strategy. Peptides were synthesized on Wang resins which were purchased from Novabiochem, and HBTU was used as activator, DIEPA as base. Double coupling was performed with microwave under 50°C for 10min. The deblock mixture was 20 % piperidine in DMF. Cocktail of TFA/TIS/Dodt/H_2_O (92.5:2.5:2.5:2.5) was used to cleave peptides off the resin. Crude peptides were purified through Xbridge Peptide BEH C18 column (10 µm, 10 mm × 250 mm) and analyzed by Eclipse XDB-C18 (5 µm, 4.6 mm × 250 mm) column. The purified peptides are identified by HPLC and MALDI-TOF MS (Figure S1). The peptides were dissolved in ddH_2_O at a concentration of 10 mM and stored at -80°C. Wang resins were purchased from Novabiochem, and the Fmoc protected amino acids were purchased from Chempep.

***2.3 Enzymatic synthesis of glycopeptide with OGT***

The reactions were performed at 37°C for 2 h, in a total volume of 100 μL solutions containing 1 mM peptide, 3 mM UDP-GlcNAc analogs, 50 ug ncOGT in buffer (50 mM Tris-HCl, pH 7.5, 10 mM Mg^2+^). The reaction mixtures were boiled for 10 min and centrifuged at 12, 000 g for 30 min to remove the enzyme. The reaction mixture (100 μL) was diluted with 100 μl de-ionized water and ready for HPLC separation.

Each sample was detected on an Agilent HPLC equipped with an Eclipse XDB-C18 Column (5 μm, 9.4*250 mm). The separation was carried out at a mobile phase gradient (mobile phase A: 100 % H_2_O, 0.1 % TFA; mobile phase B: 100 % ACN and 0.1 % TFA) of 5 % to 35 % B in 30 min at a flow rate of 2.5 ml/min. The detection wavelength was set at 220 nm. The yield was calculated through the formula Y= S1/ (S1+S2), where S1 and S2 stand for integrated areas of the product peak and substrate peak, respectively. HPLC analysis was performed in triplicate.

***2.4 OGA substrate specificity assay***

The glycopeptide peaks were collected from above HPLC seperation, lyophilized and dissolved in appropriate amounts of reaction buffer (50 mM Tris-HCI, pH 7.5, 10 mM Mg^2+^). The reactions were performed at 37°C overnight, in a total volume of 100 μL solutions containing glycopeptide and 100ug OGA. The reaction mixtures were boiled for 10 min and centrifuged at 12, 000g for 30 min to remove the enzyme before HPLC separation.

**3. Supplemental figures and tables**

**
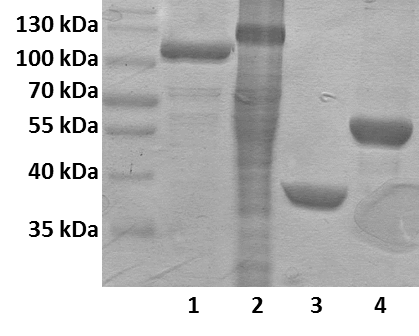
**

Fig. S1 SDS-PAGE analysis of purified enzymes. Lanes: 1. OGT; 2. OGA; 3. NahK; 4. AGX1.

Table S1 Synthesis of UDP- GlcNAc derivatives using NahK and AGX1.

**UDP-GlcNPh**

**
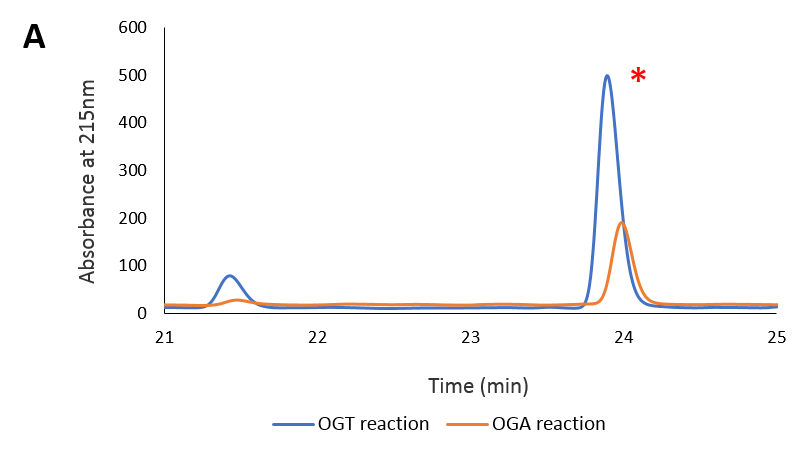

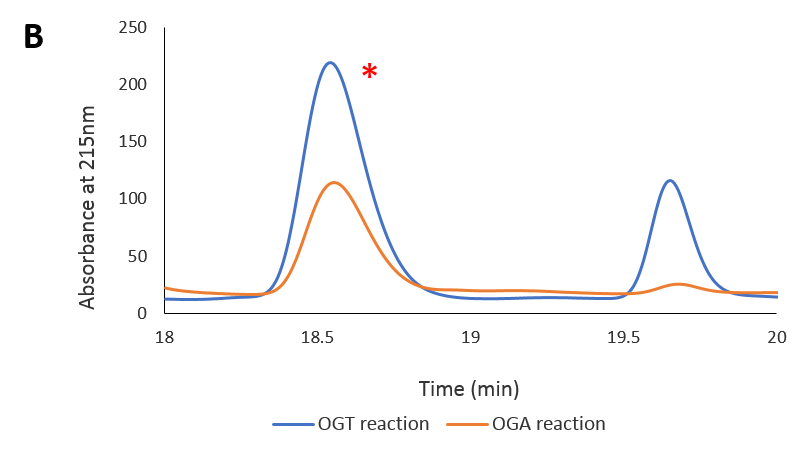

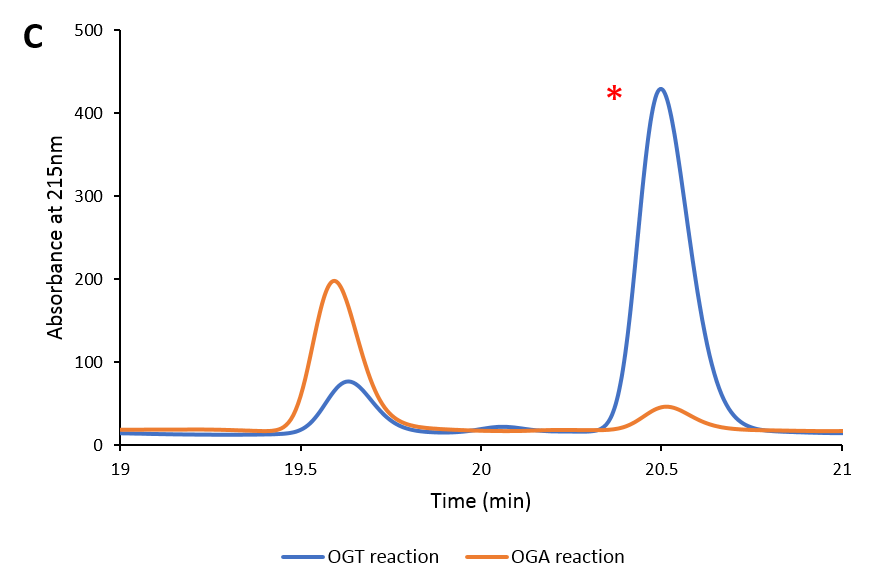
**

**
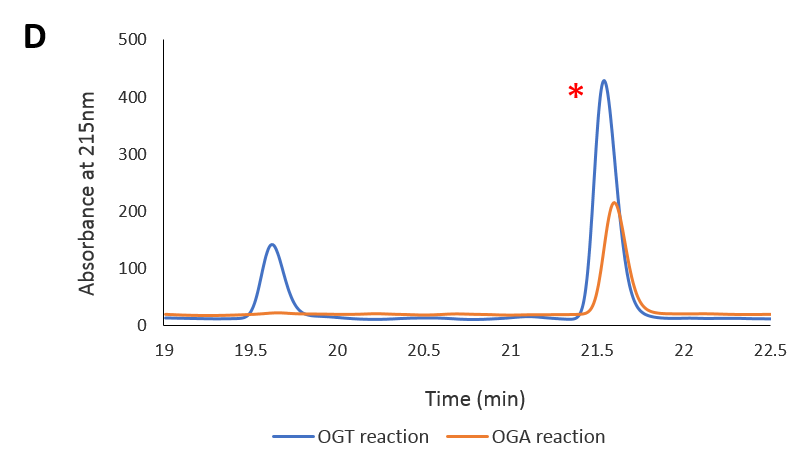
**

**
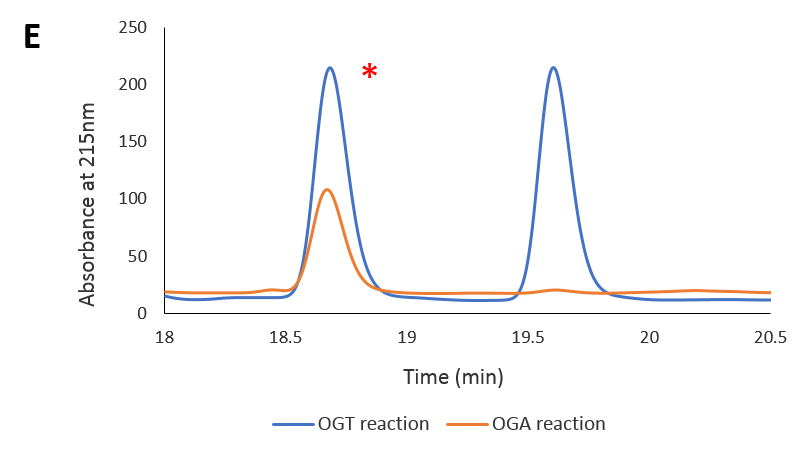
**

**
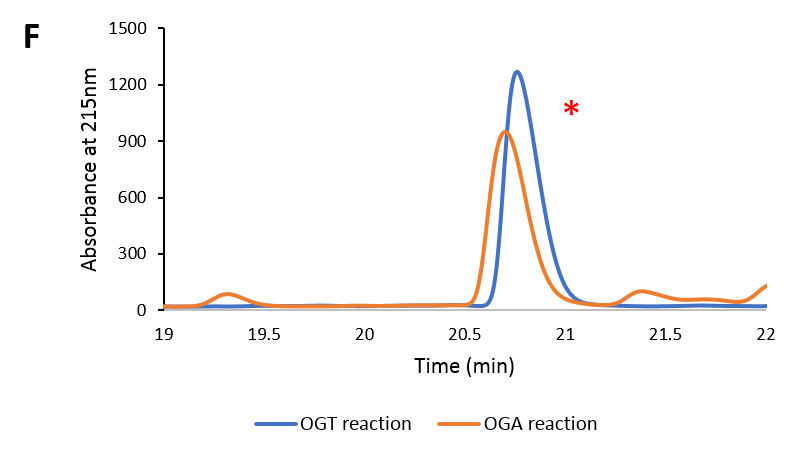
**

**
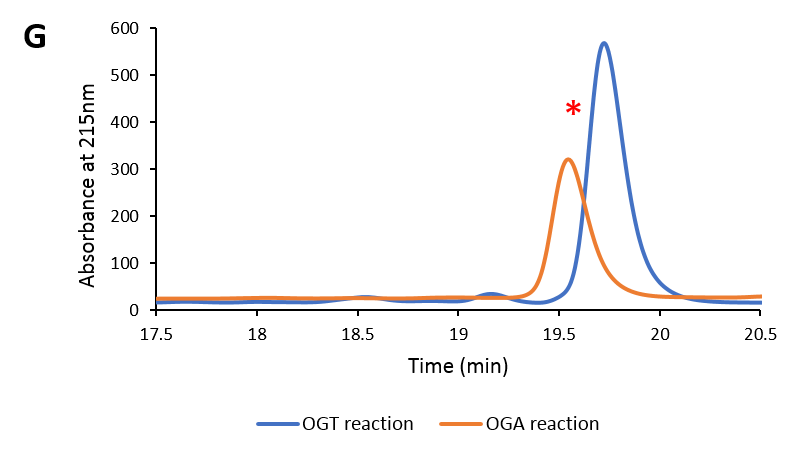
**

**
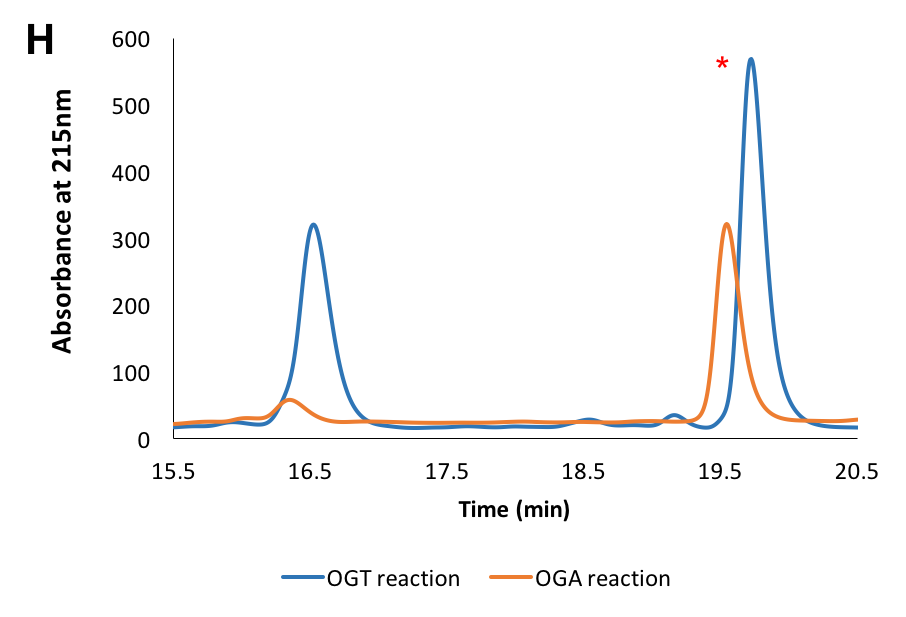
**

**
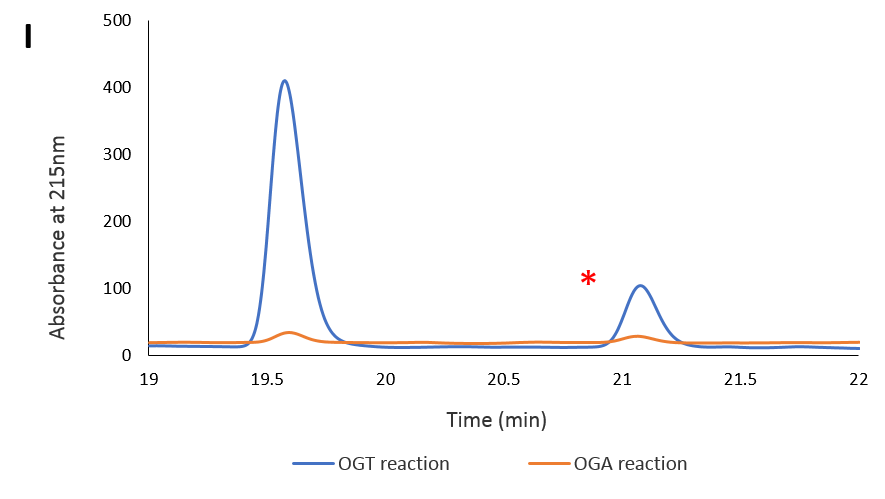
**


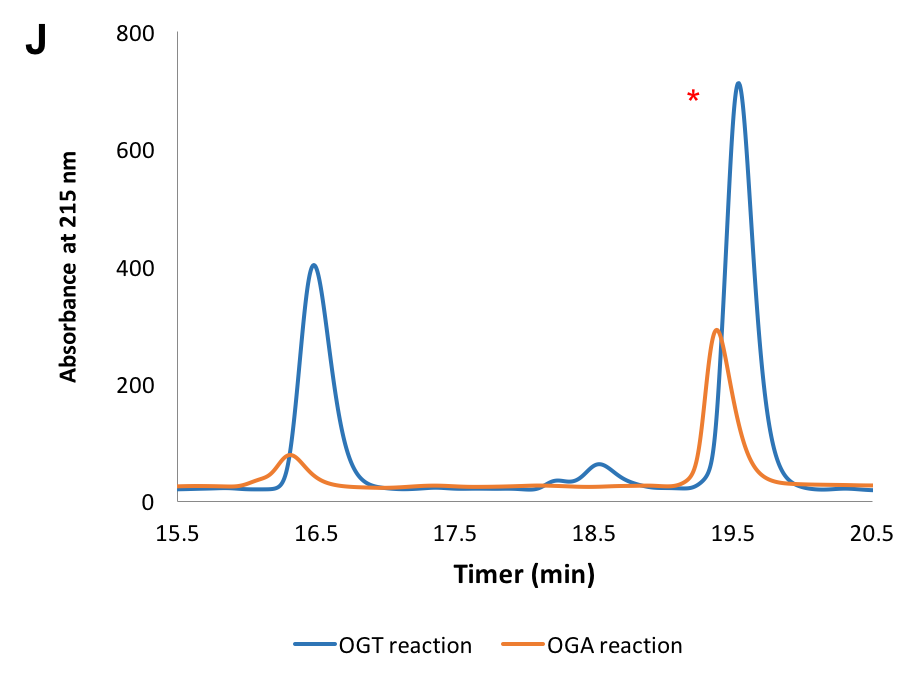


Fig.S2 HPLC file of OGT and OGA reactions. (A) UDP-GlcNPh, (B) UDP-GlcNGc, (C) UDP-GlcNAz, (D) UDP-GalNAc, (E) UDP-GlcNPr, (F) UDP-GlcNTFA, (G) UDP-4-deoxy-GlcNAz, (H) UDP-4-OMe GlcNAc, (I) UDP-6-N_3_-GlcNAc, (J) UDP-6-deoxy-GlcNAc as sugar donor. * represents the glycopeptide peak.


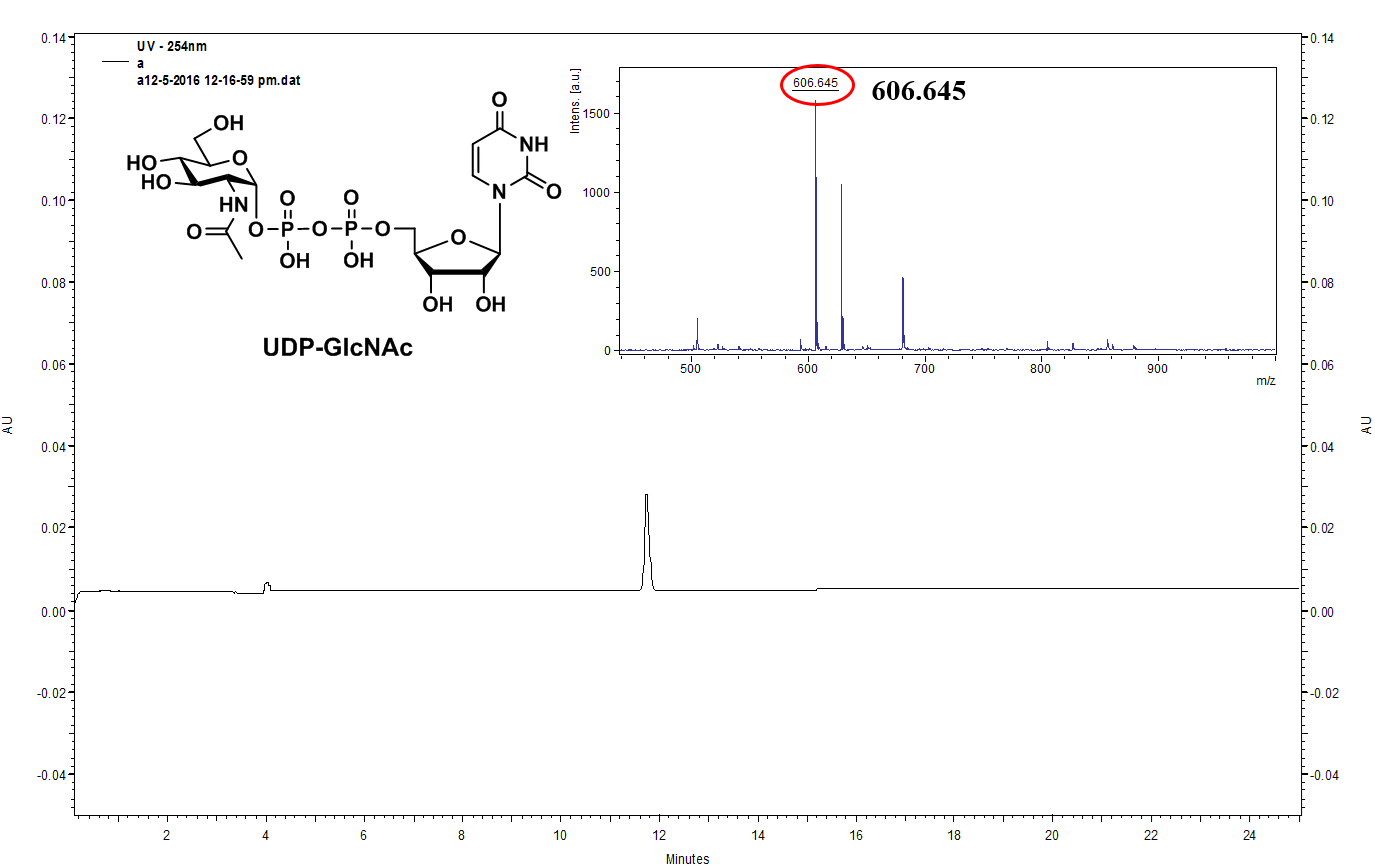


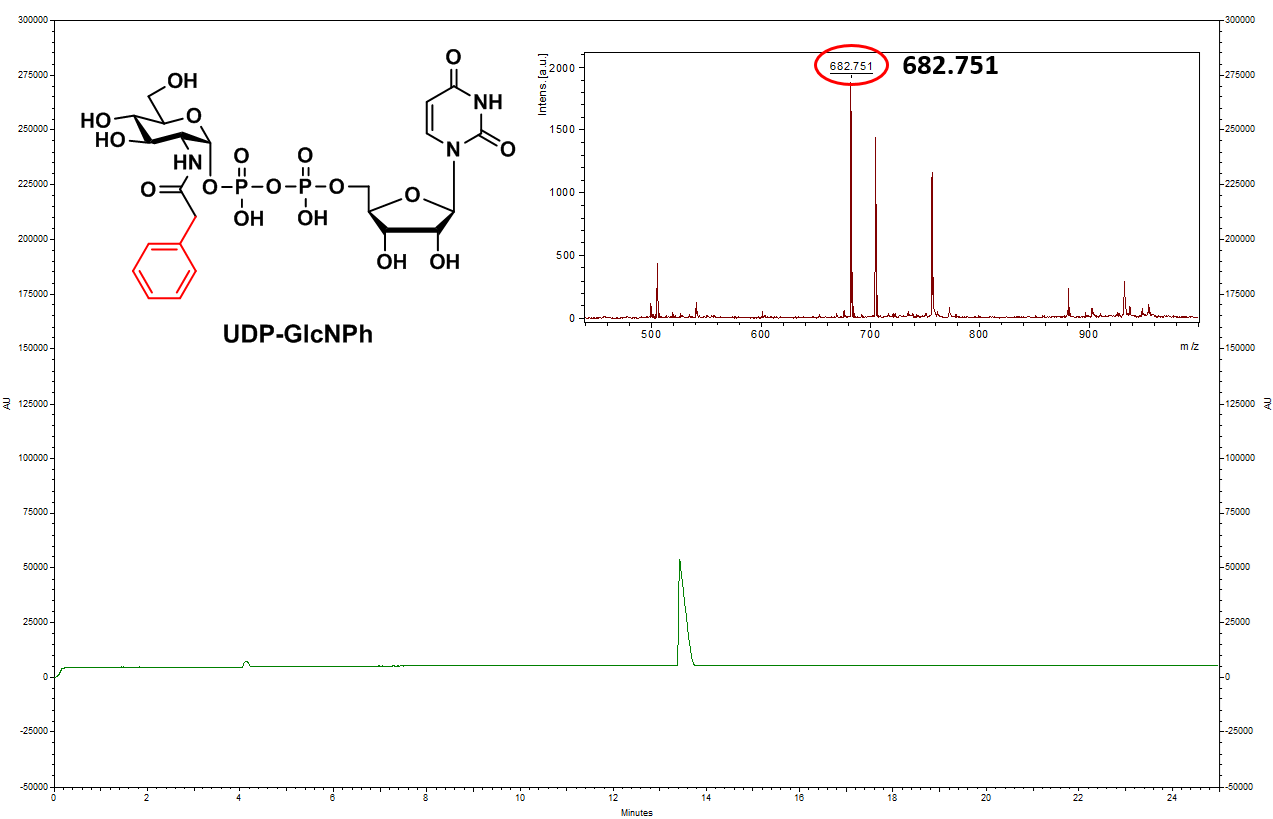


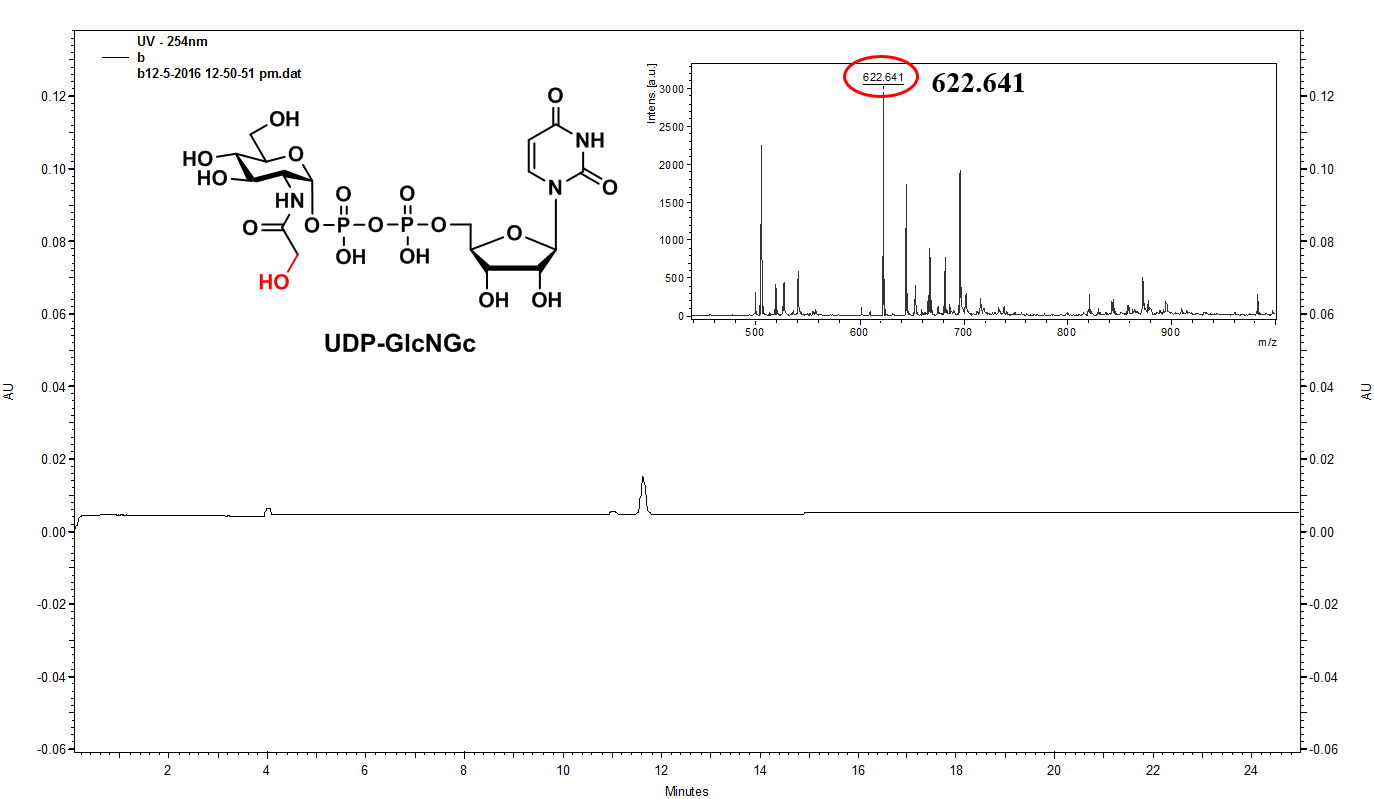


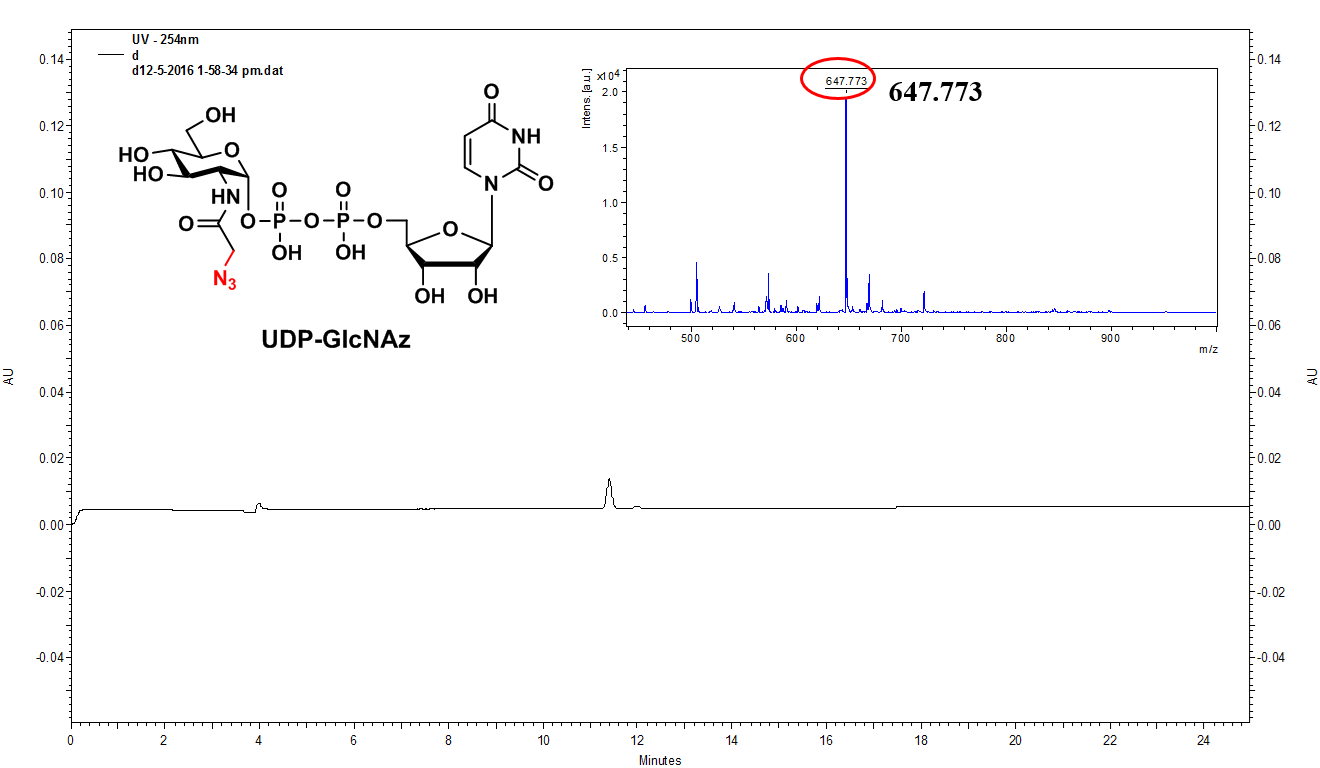


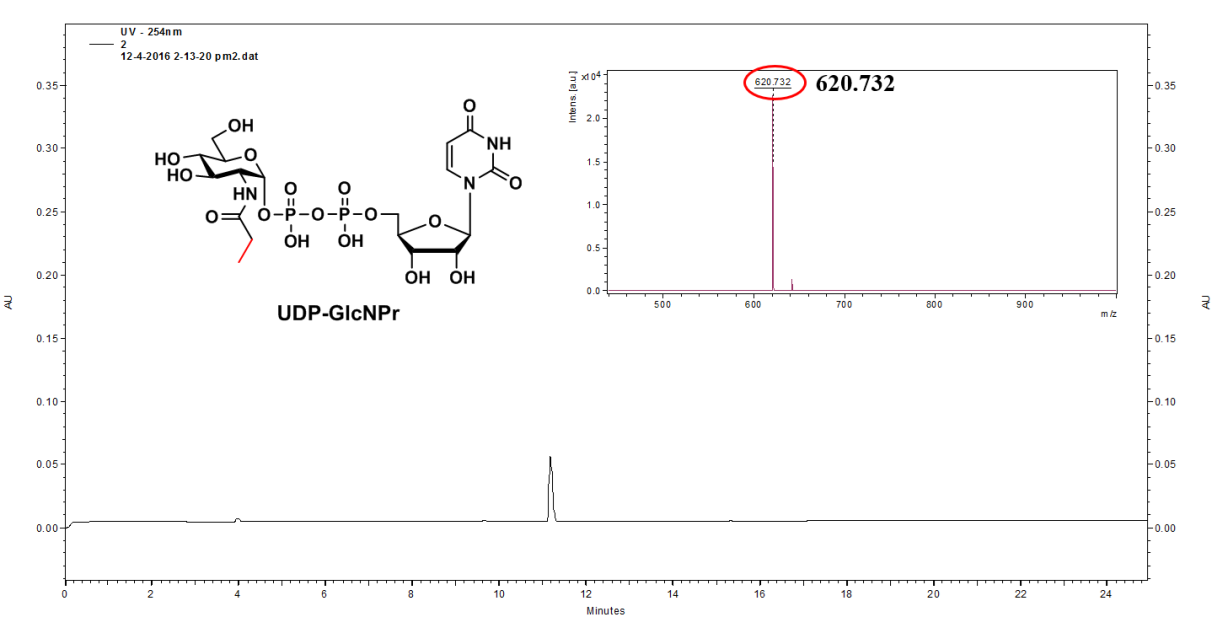


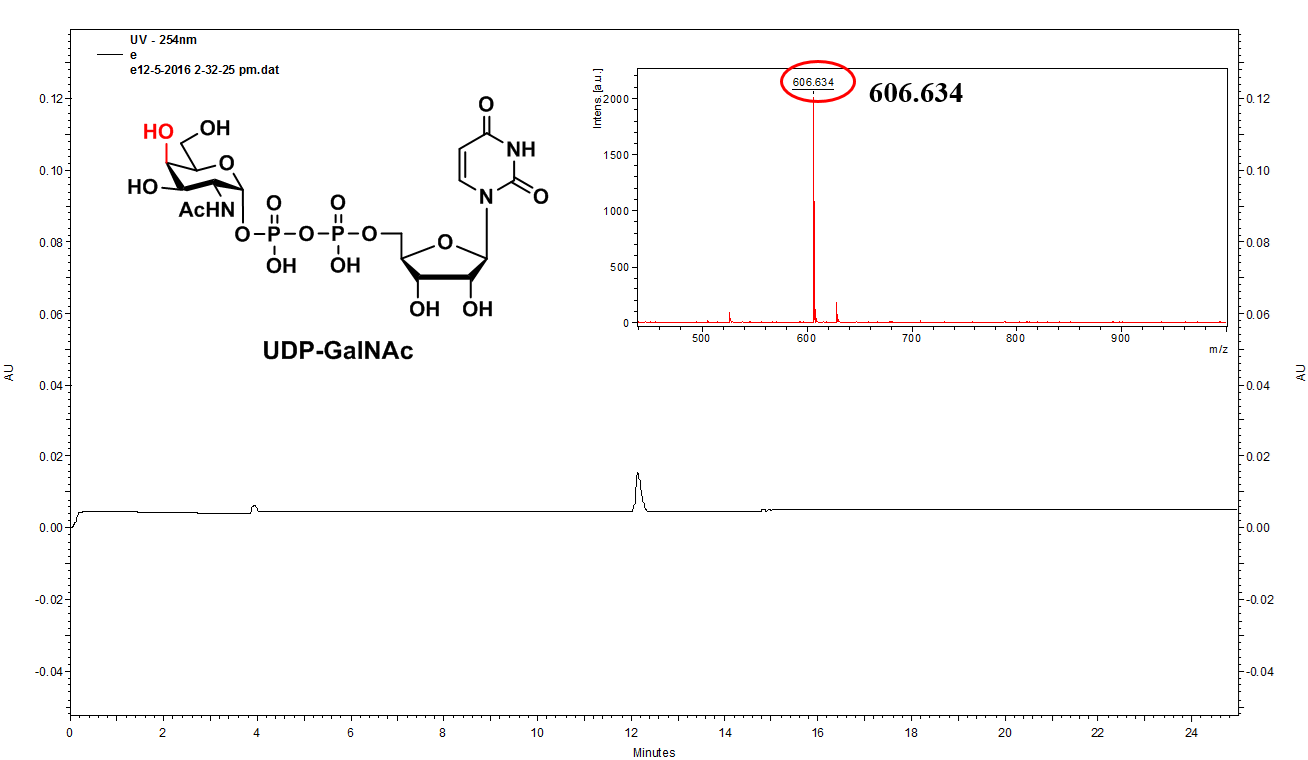


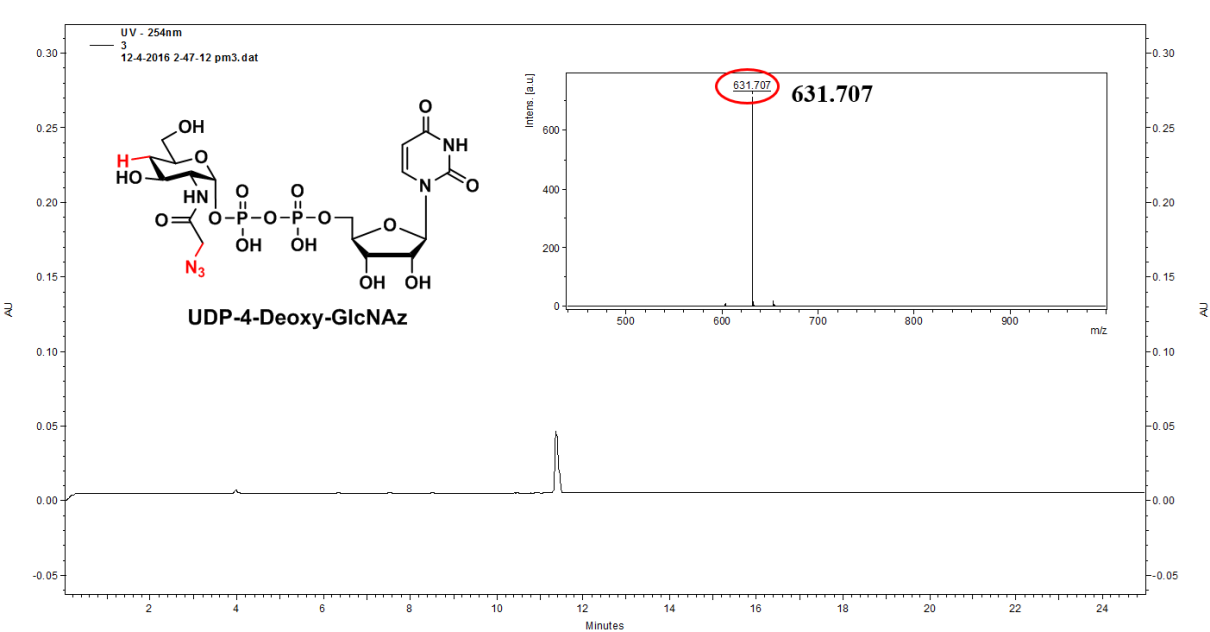


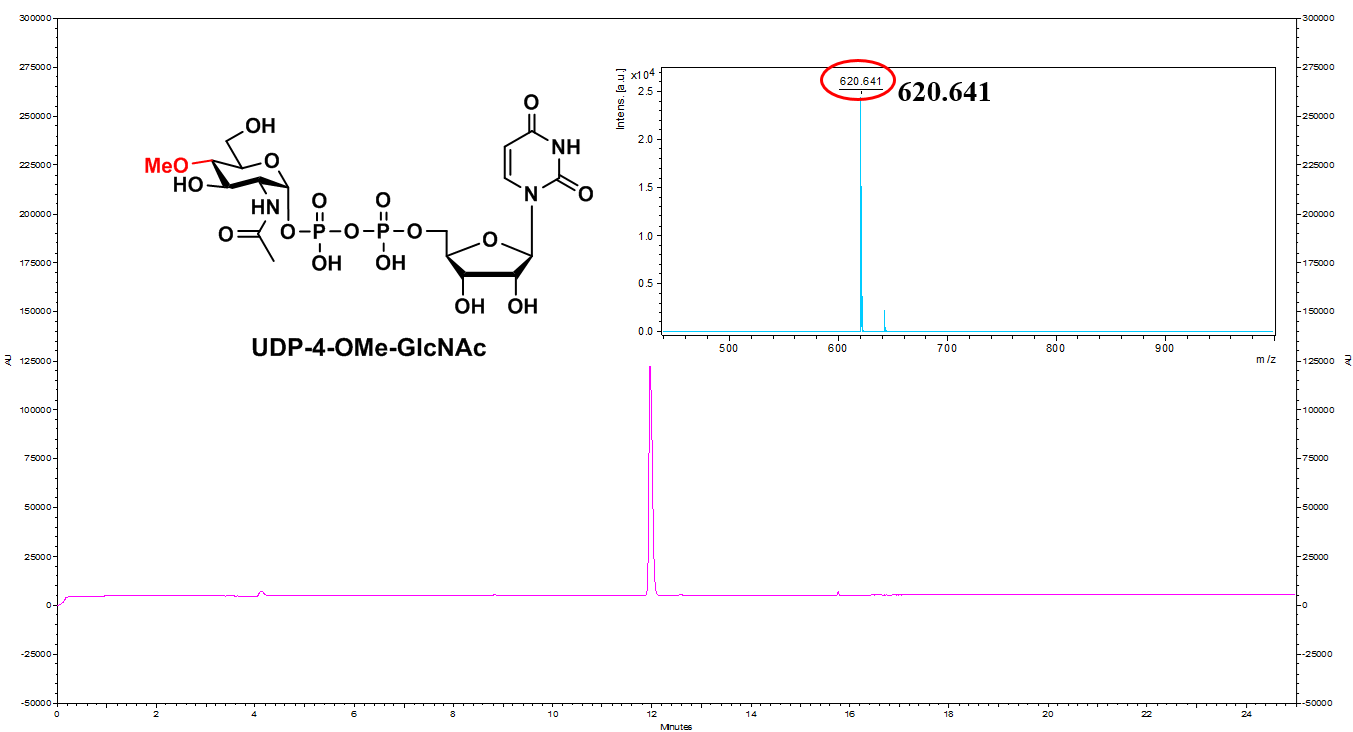


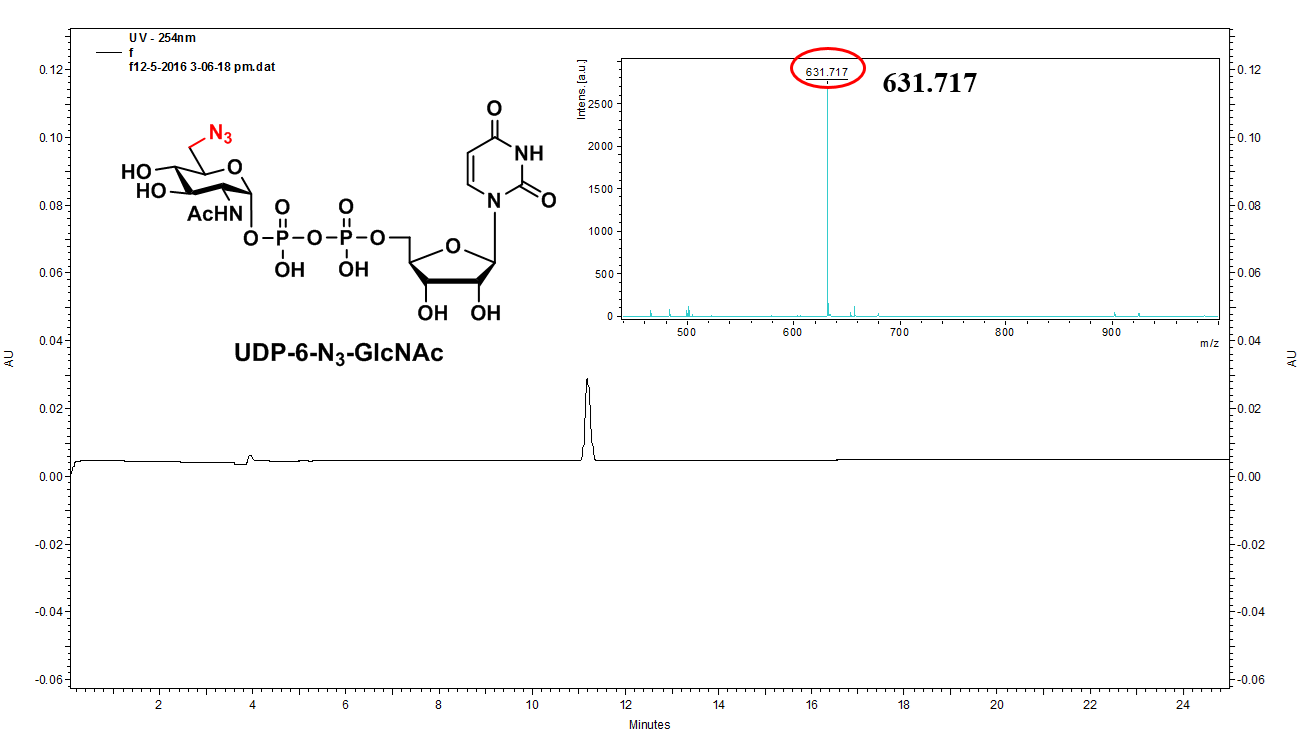


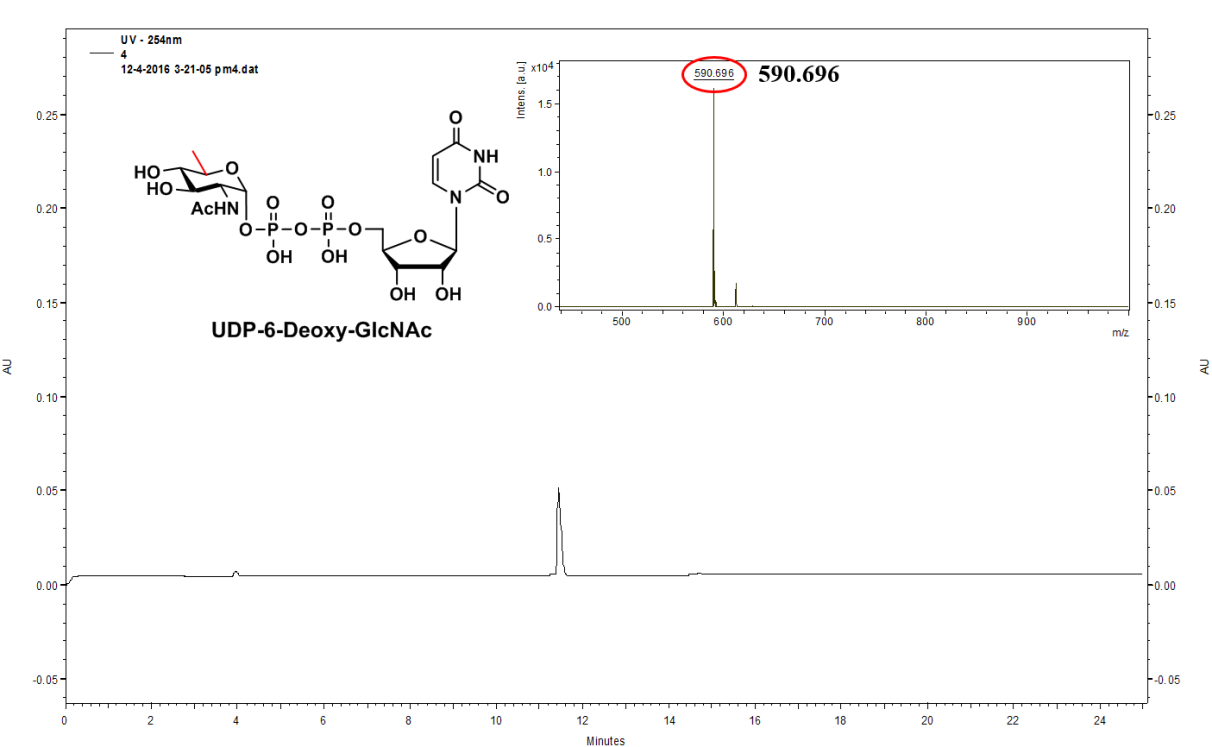


Fig. S3 Characterization of UDP sugars using MALDI and capillary electrophoresis (CE).

Fig. S4 (A) The structure of peptide used in this study; (B) the MALDI-MS identification; (C) the purity check by HPLC. HPLC condition: elute by 5 %-35 % acetonitrile with 0.1 % TFA for 30 min at 2.5 ml/min flow rate.


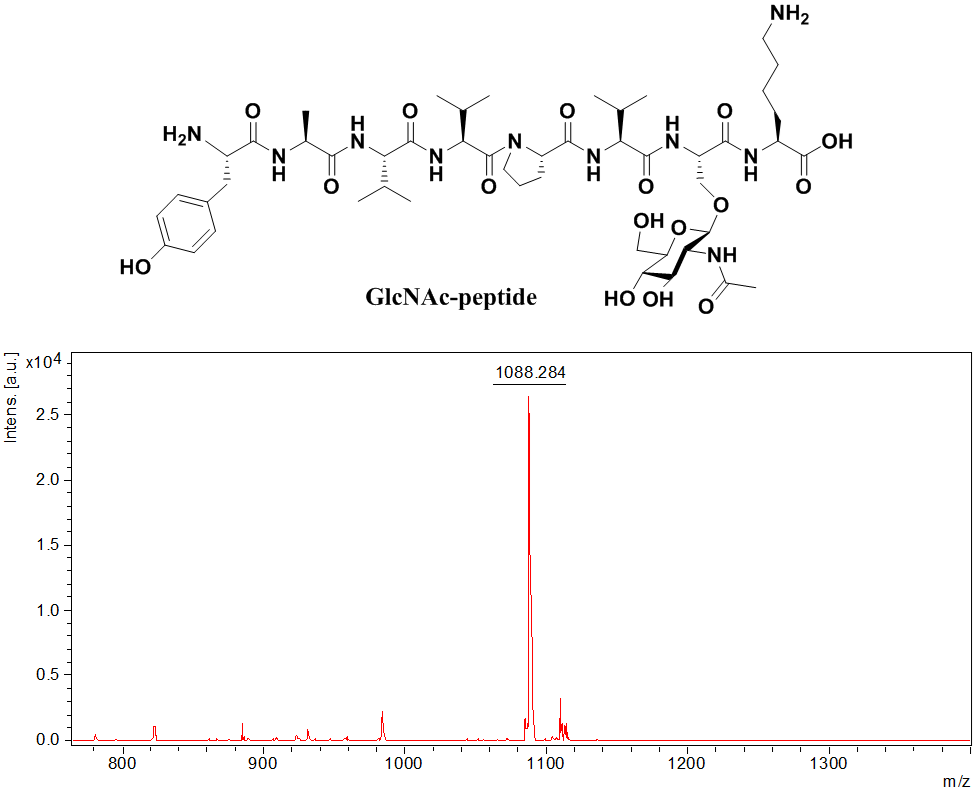

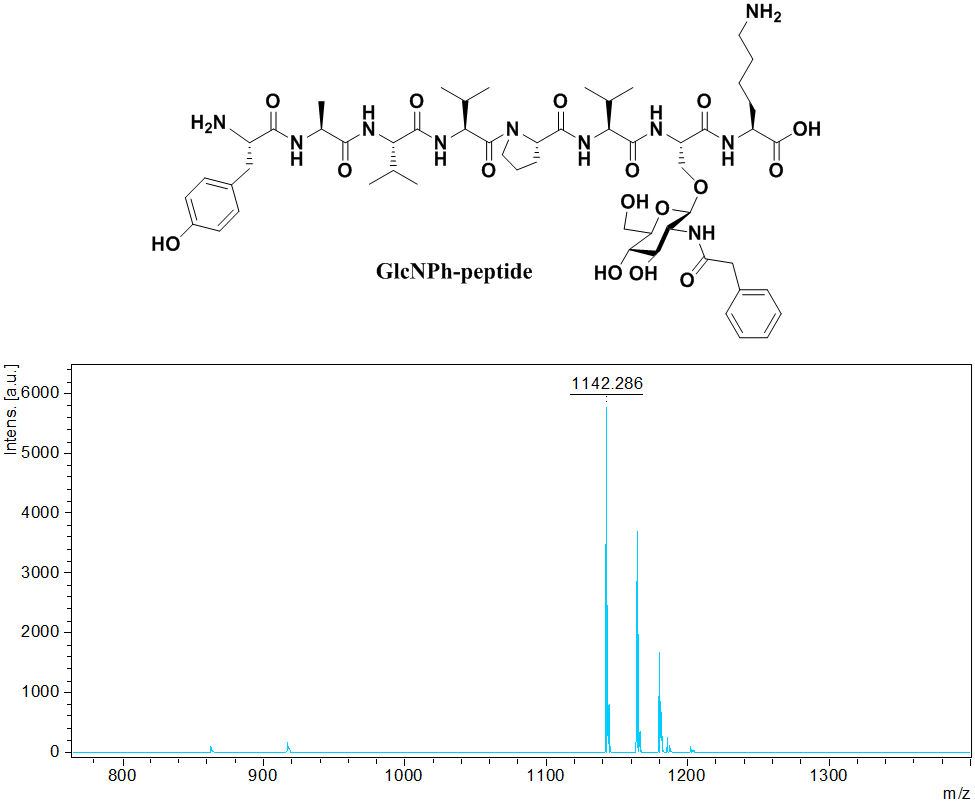


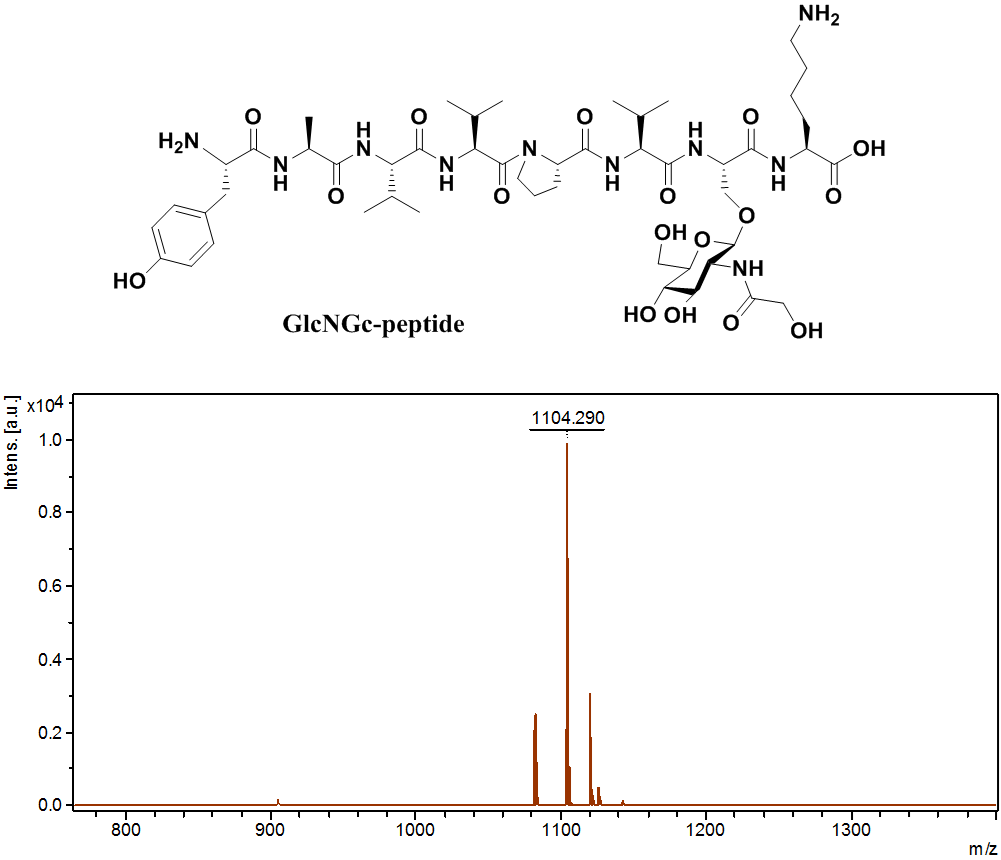


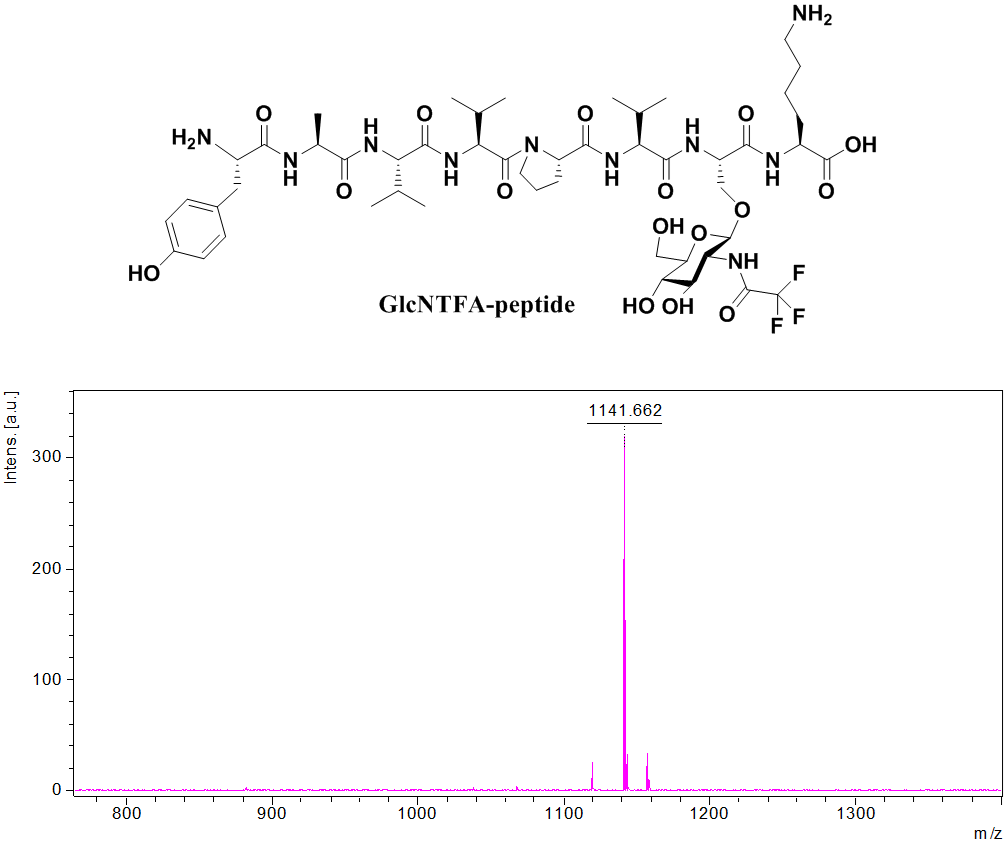


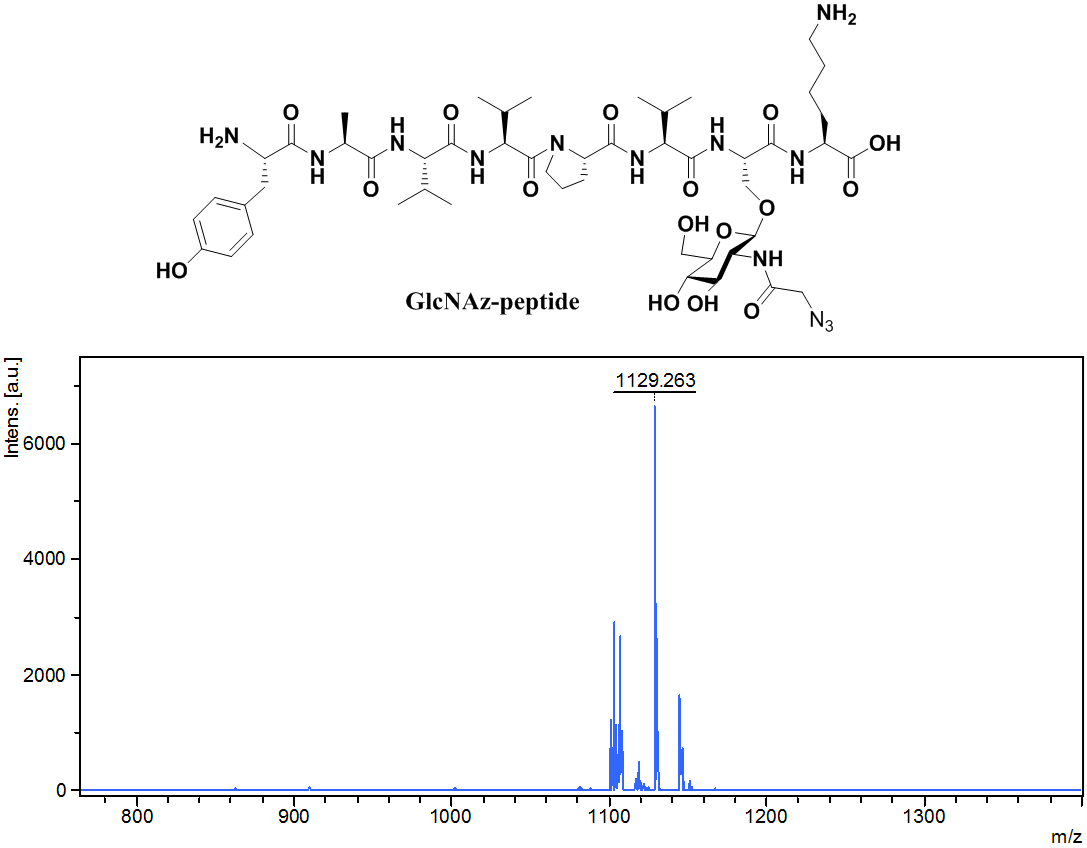


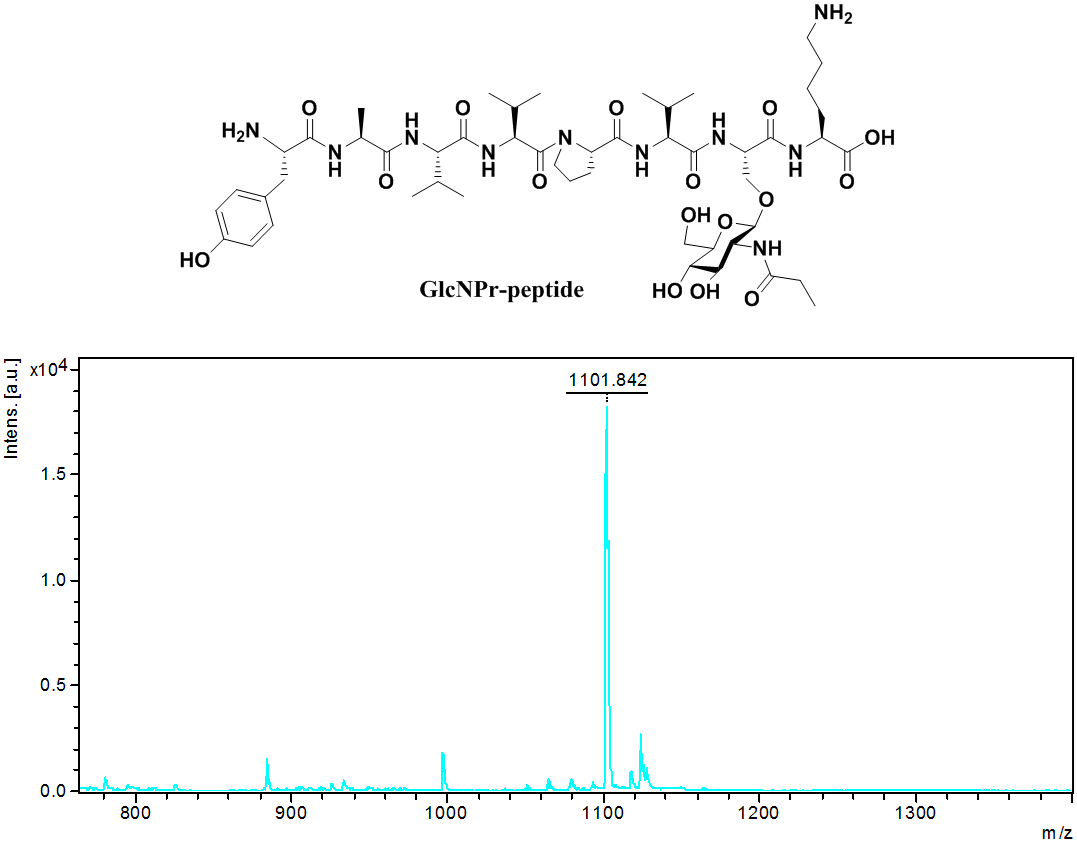


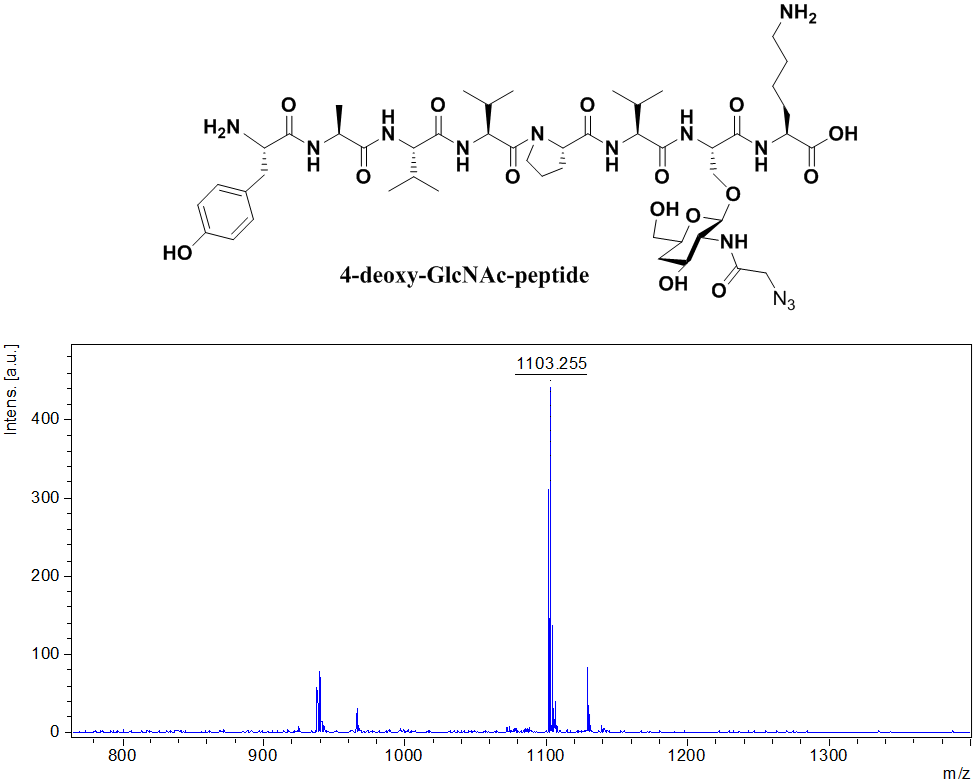


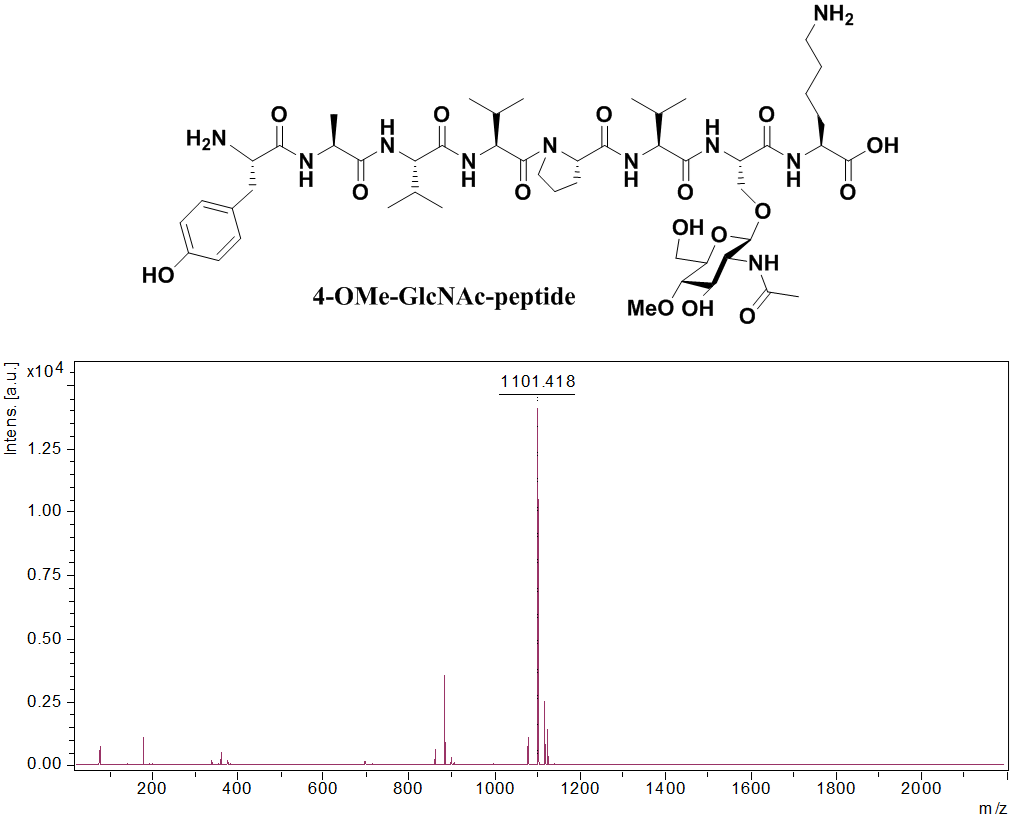


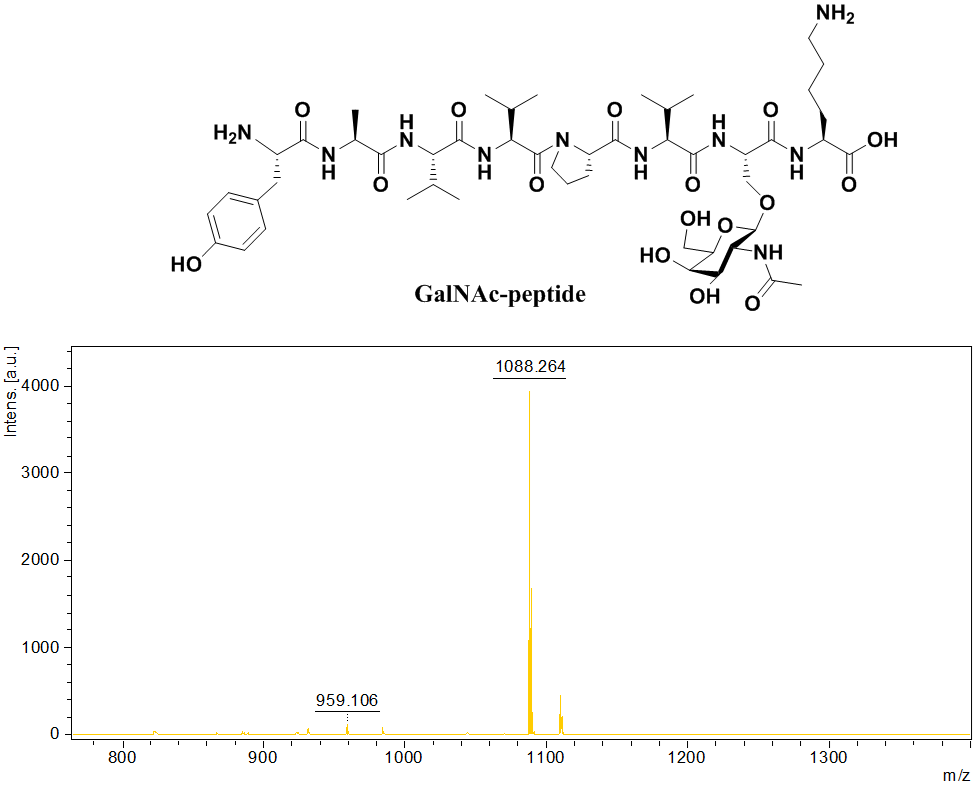


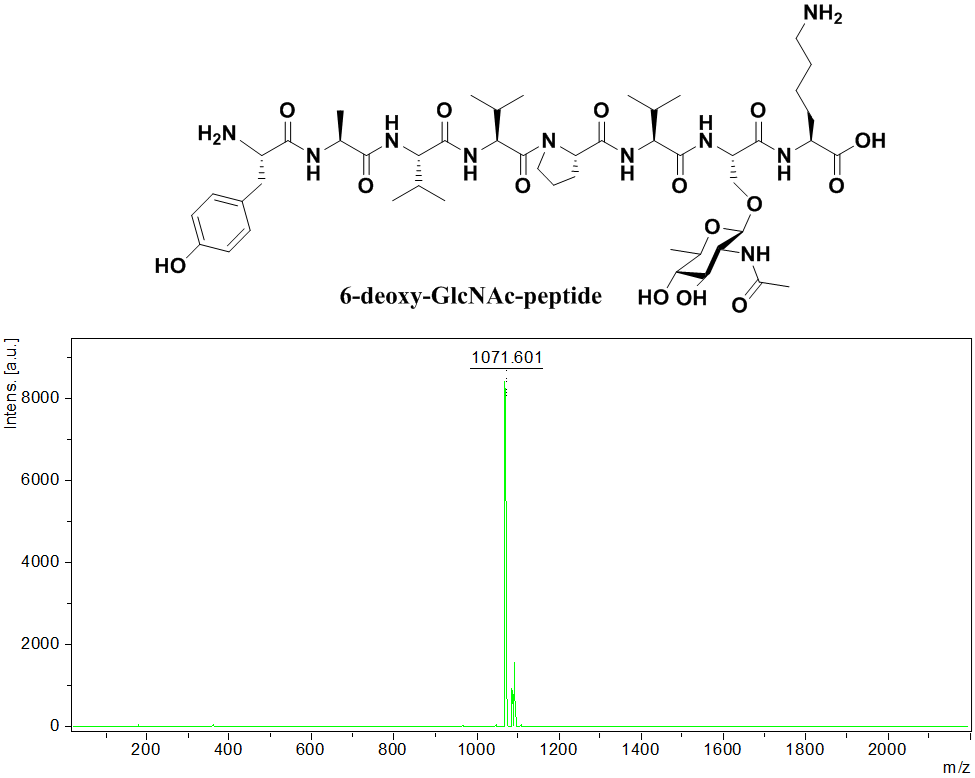


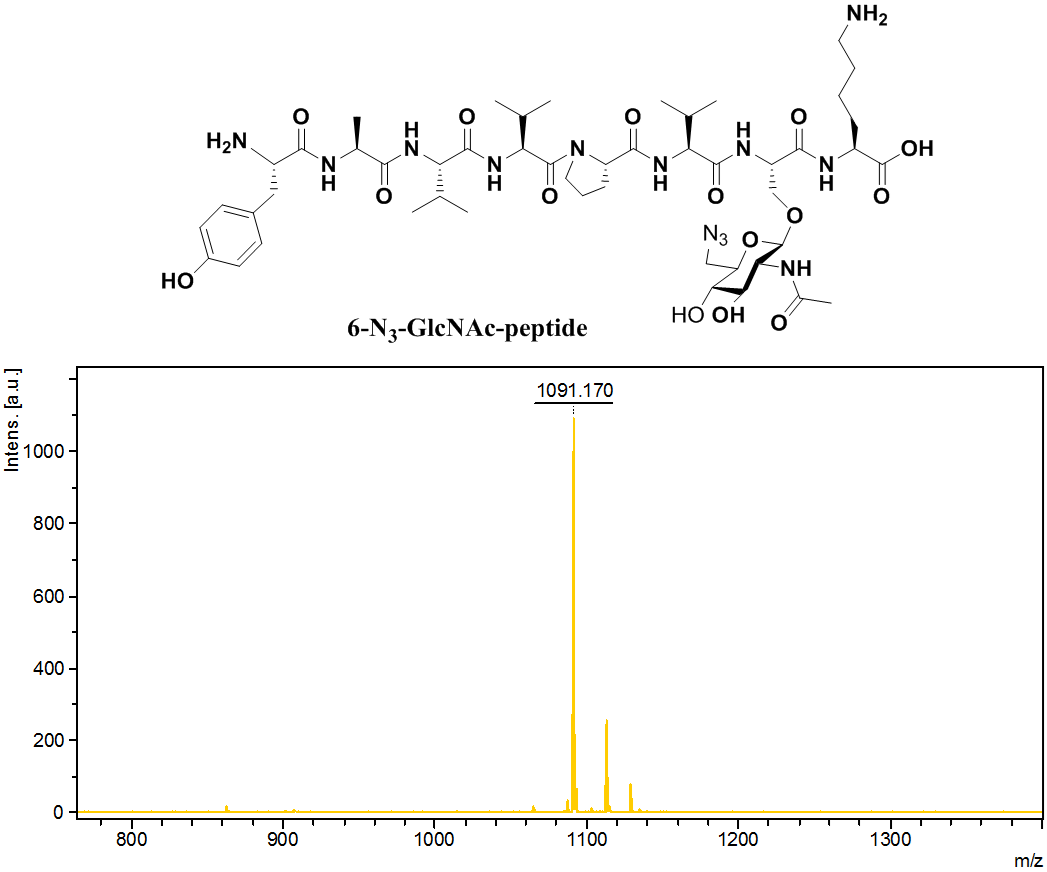


Fig. S5 Characterization of glycopeptide derivatives using MALDI.

**4. NMR spectrums of GlcNAc derivatives**


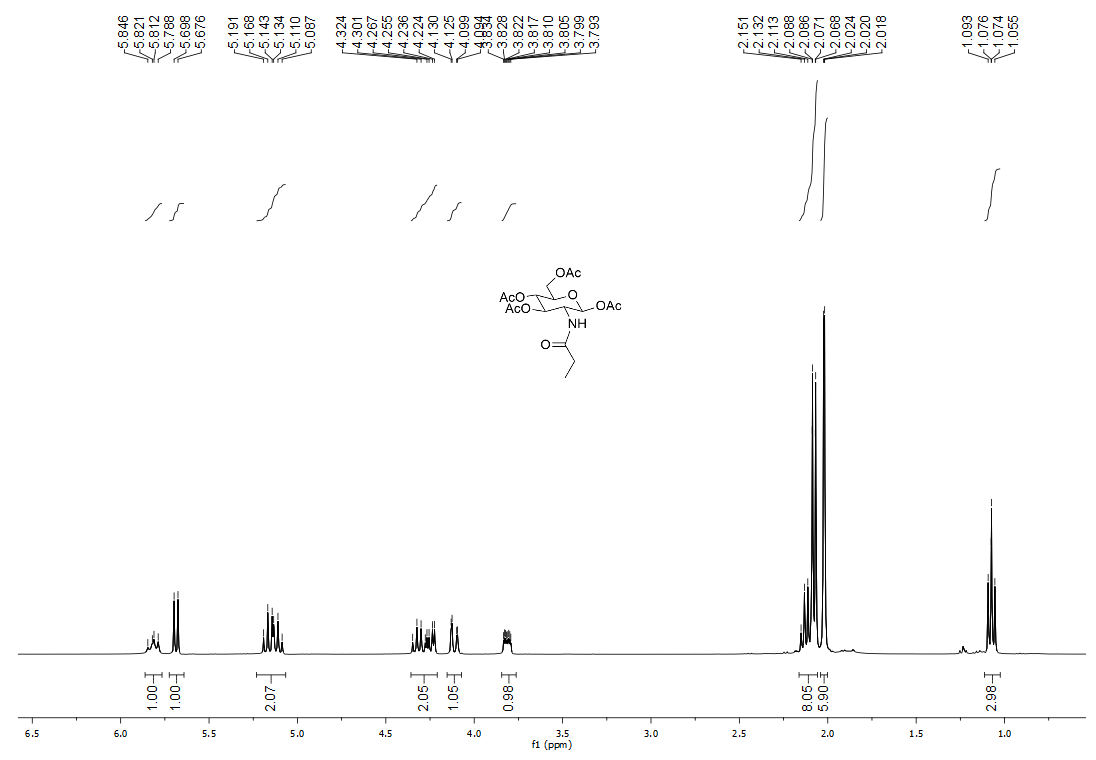


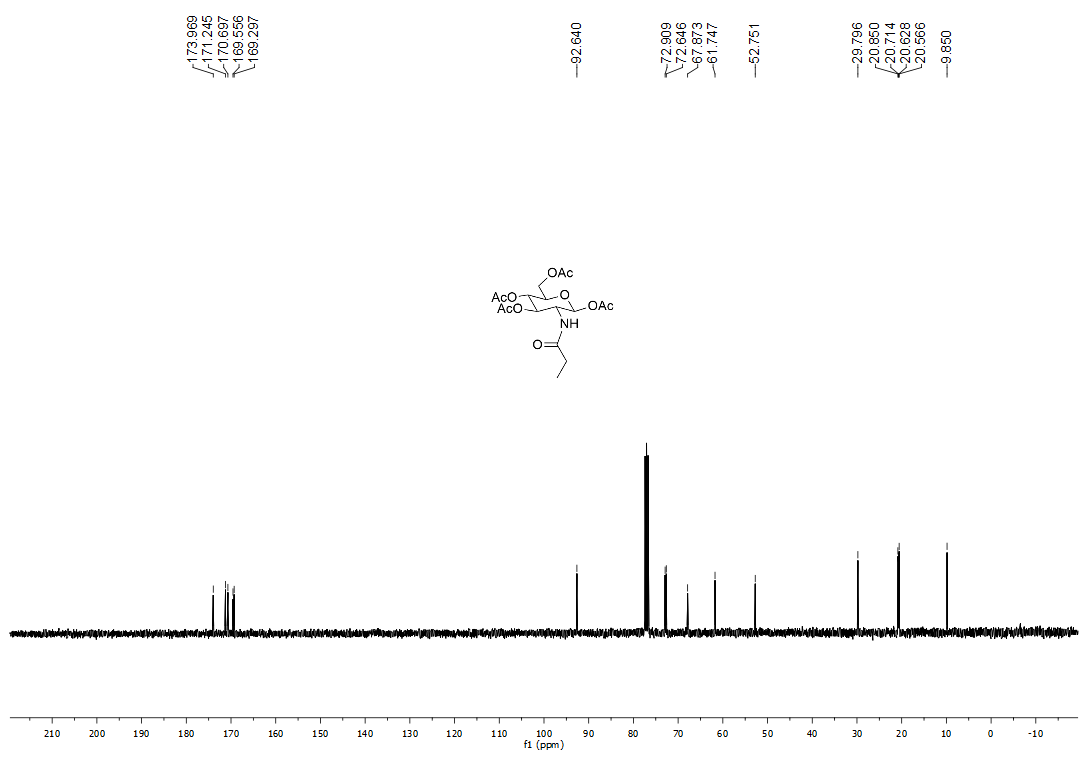


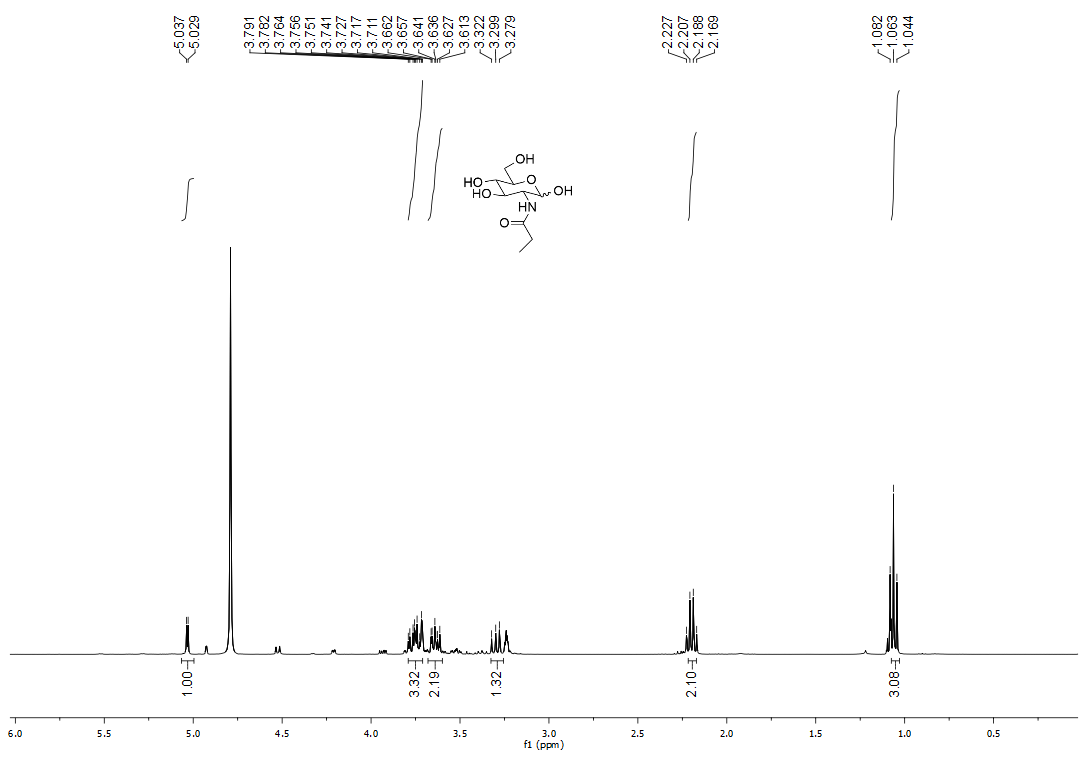


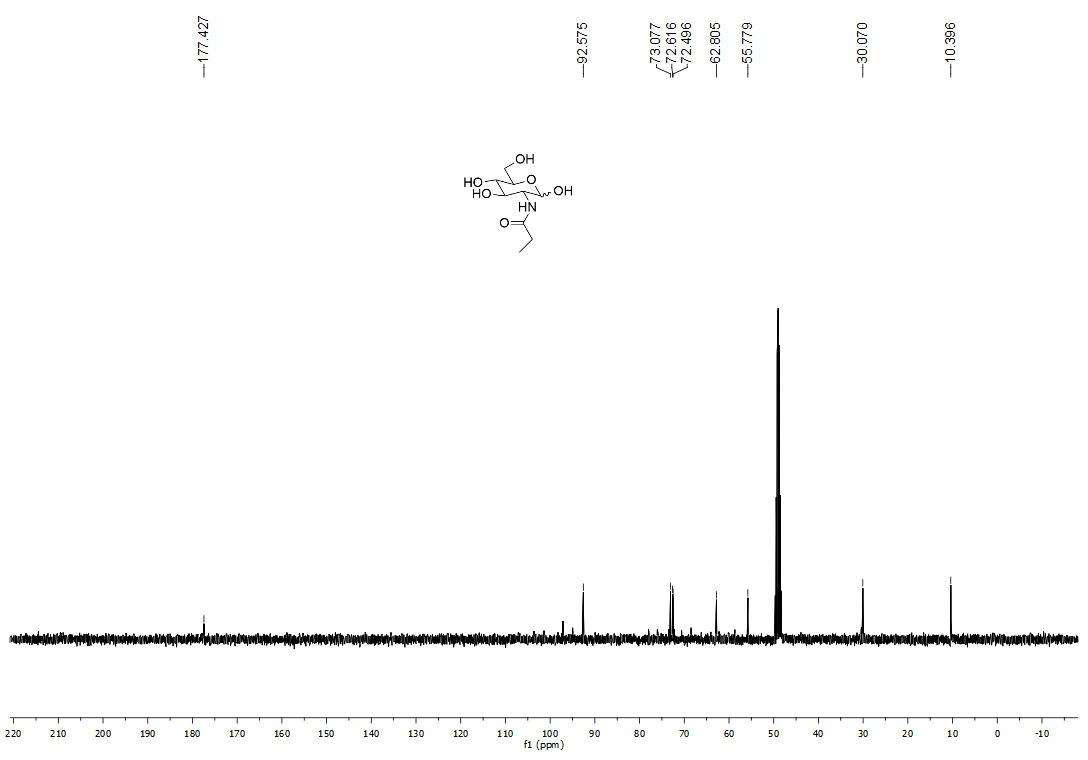


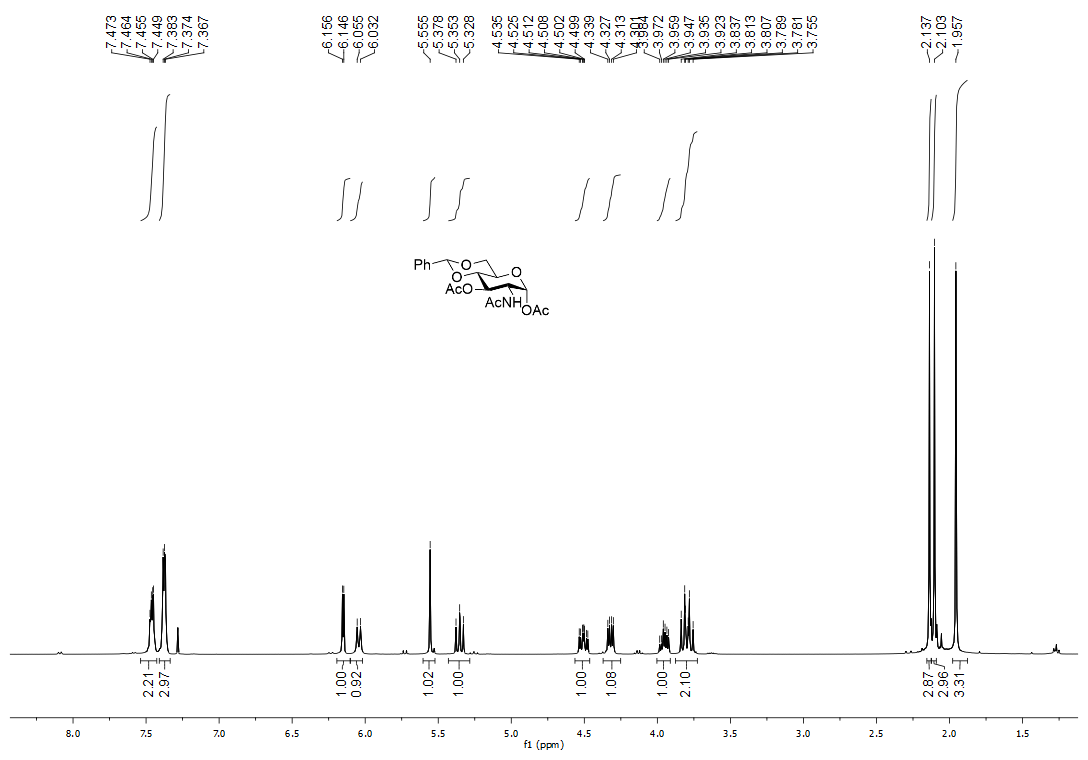


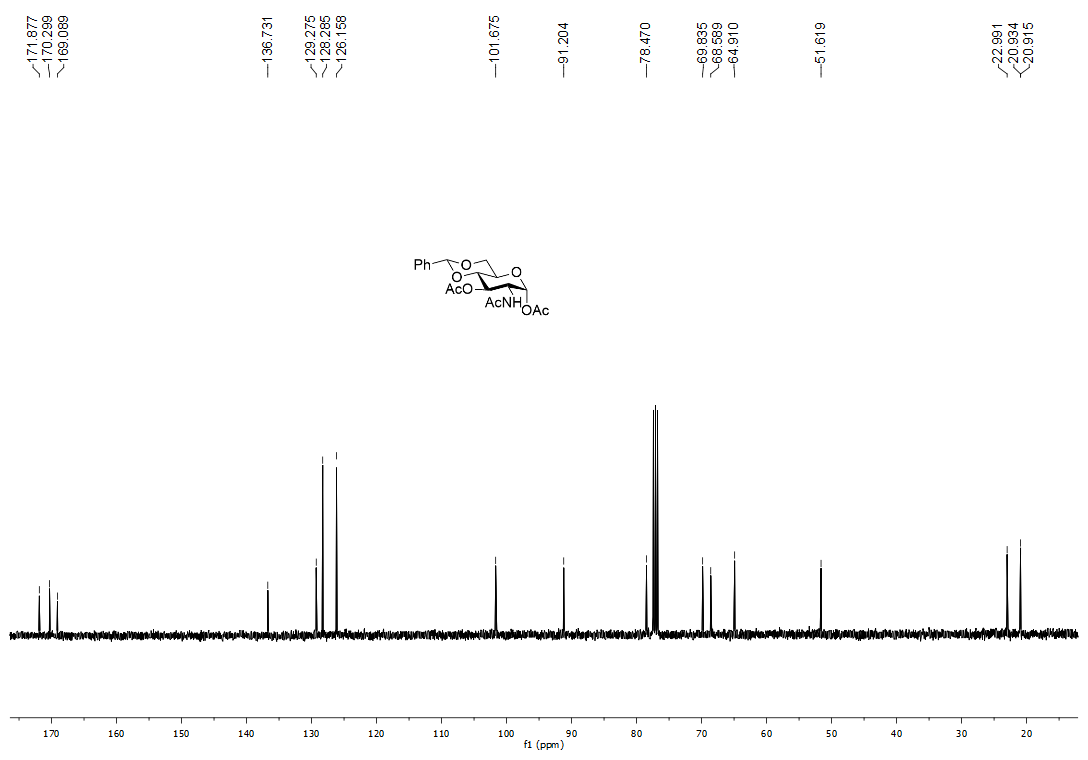


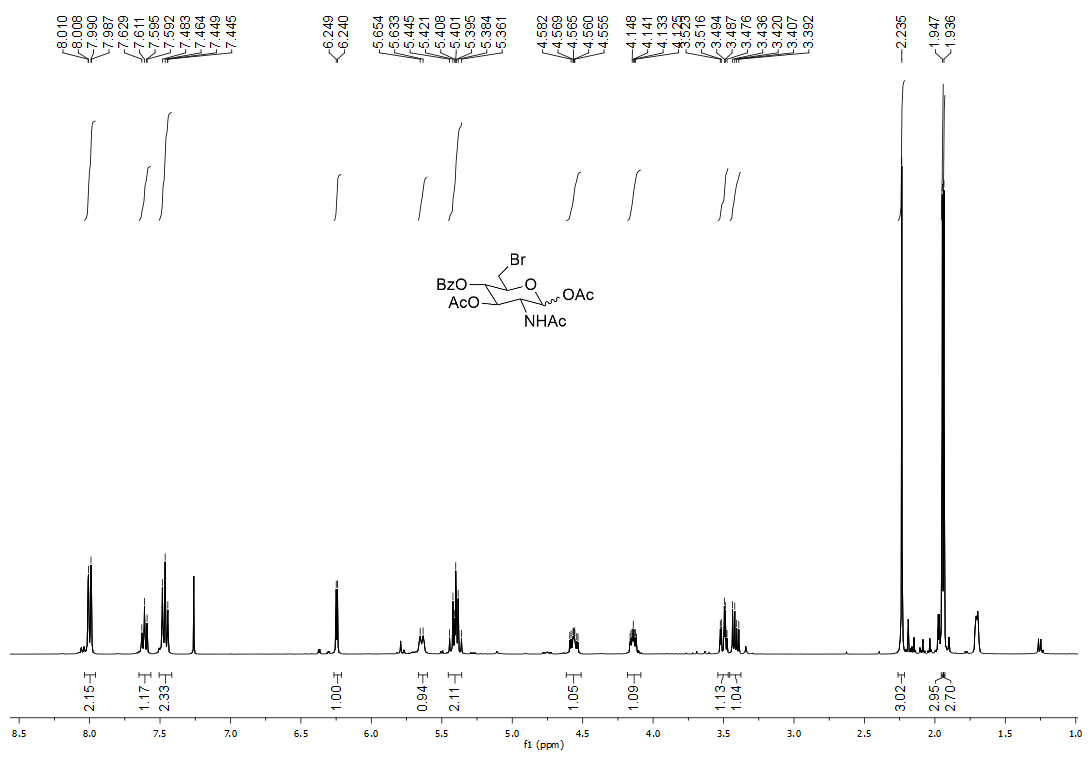


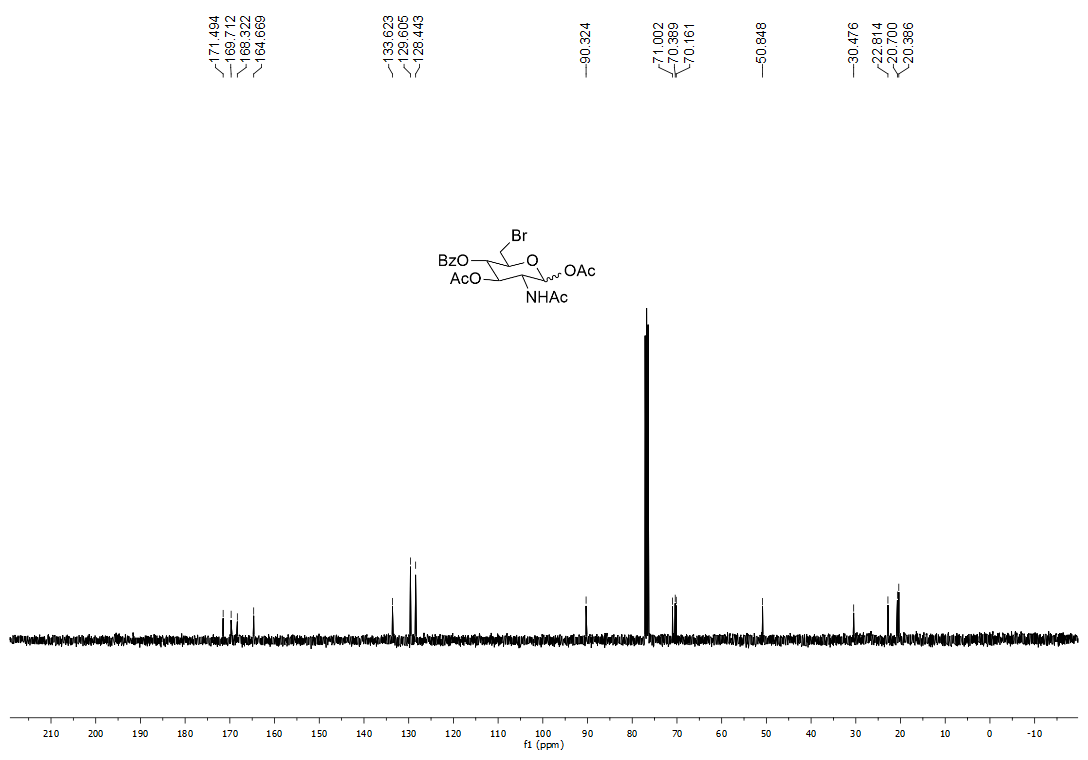


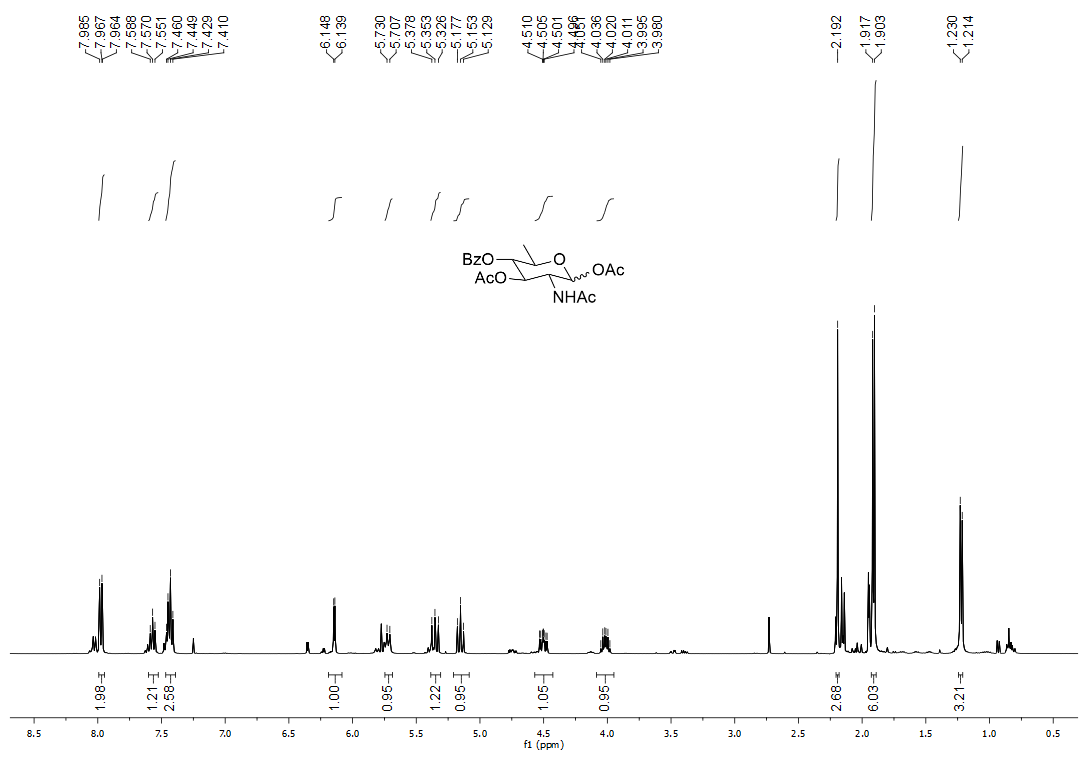


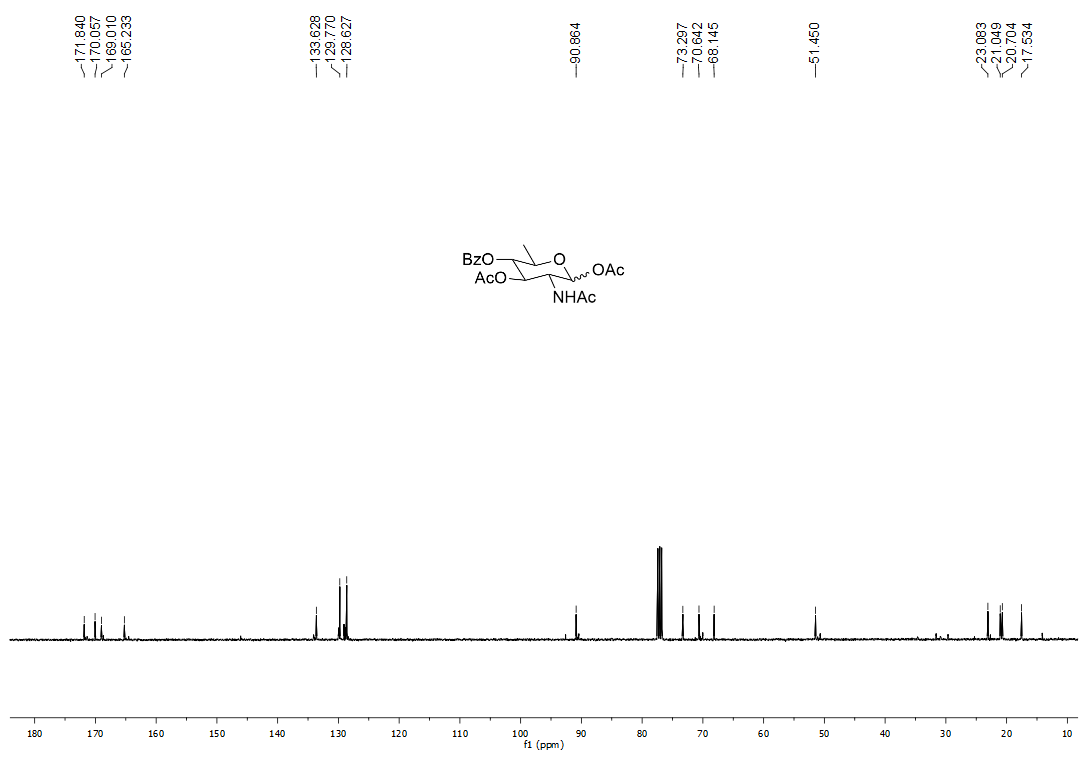


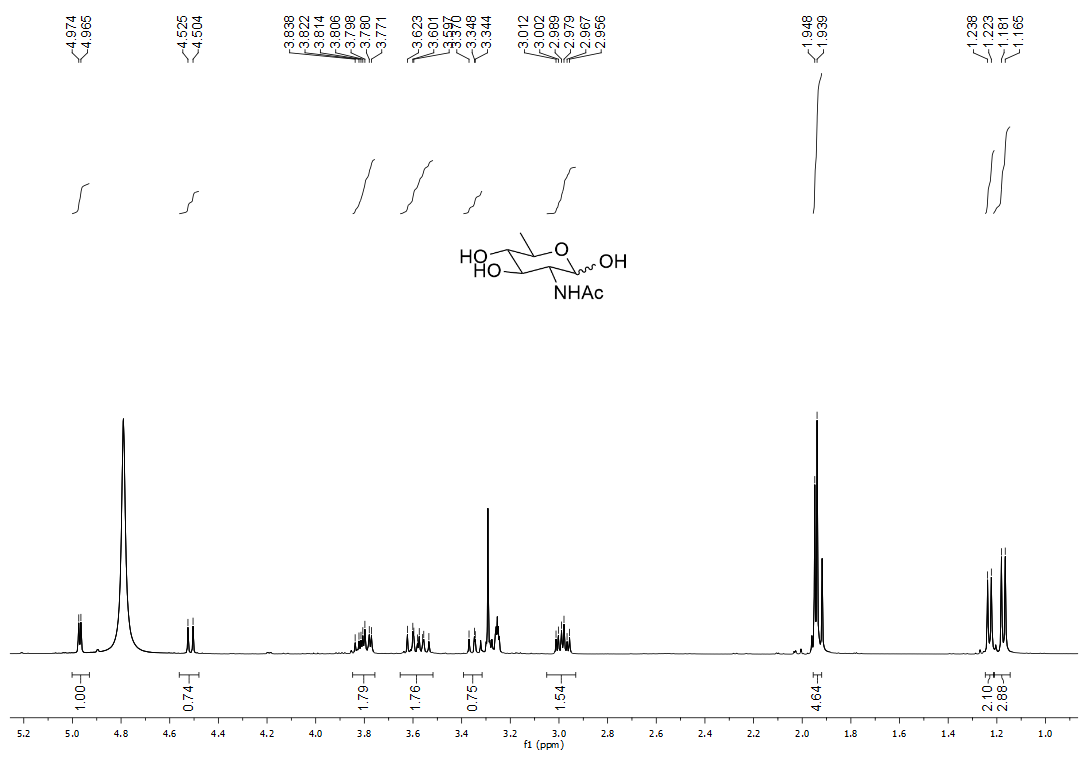


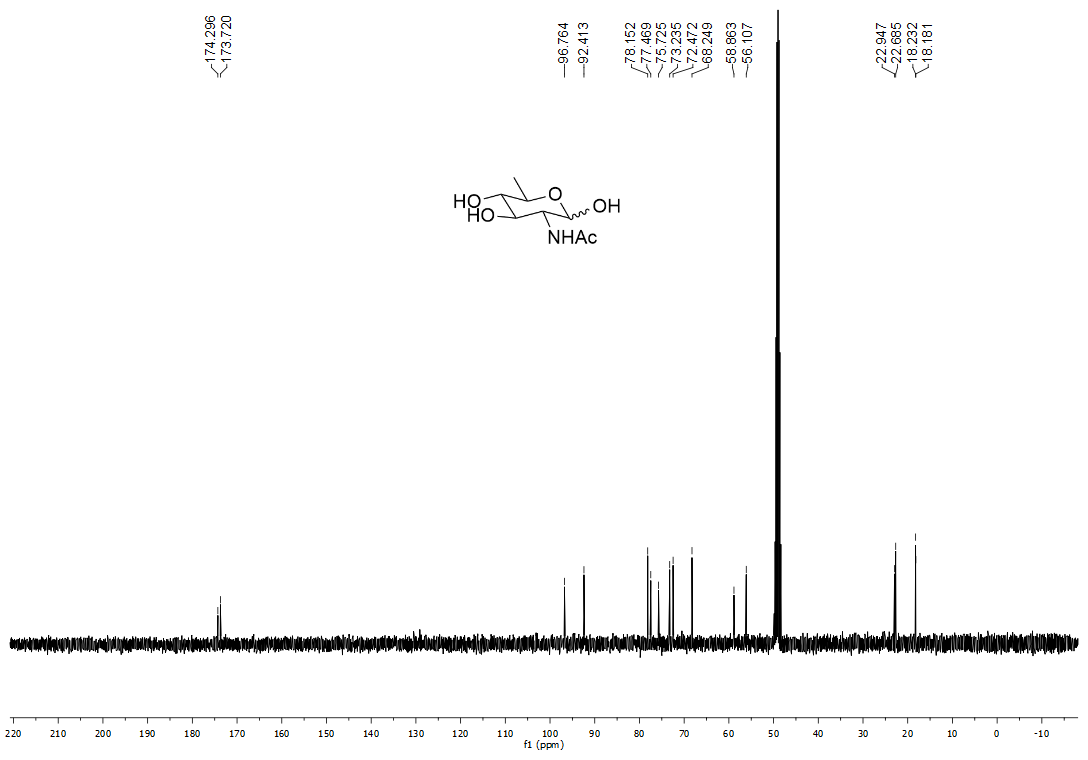


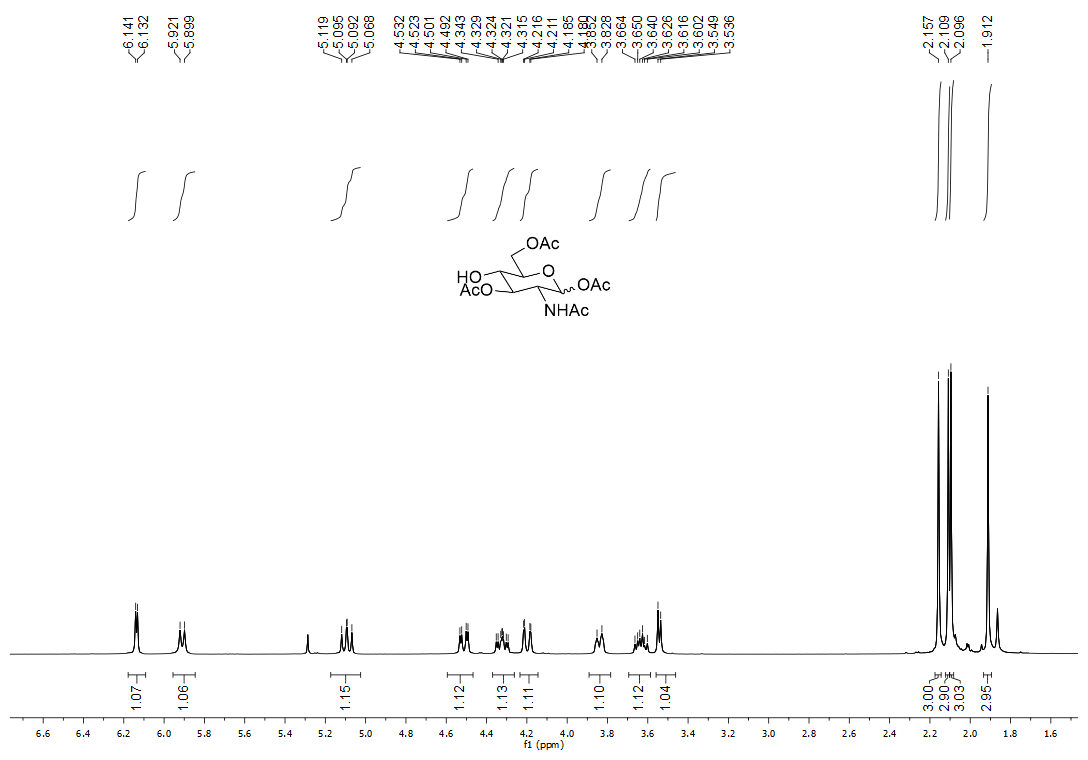


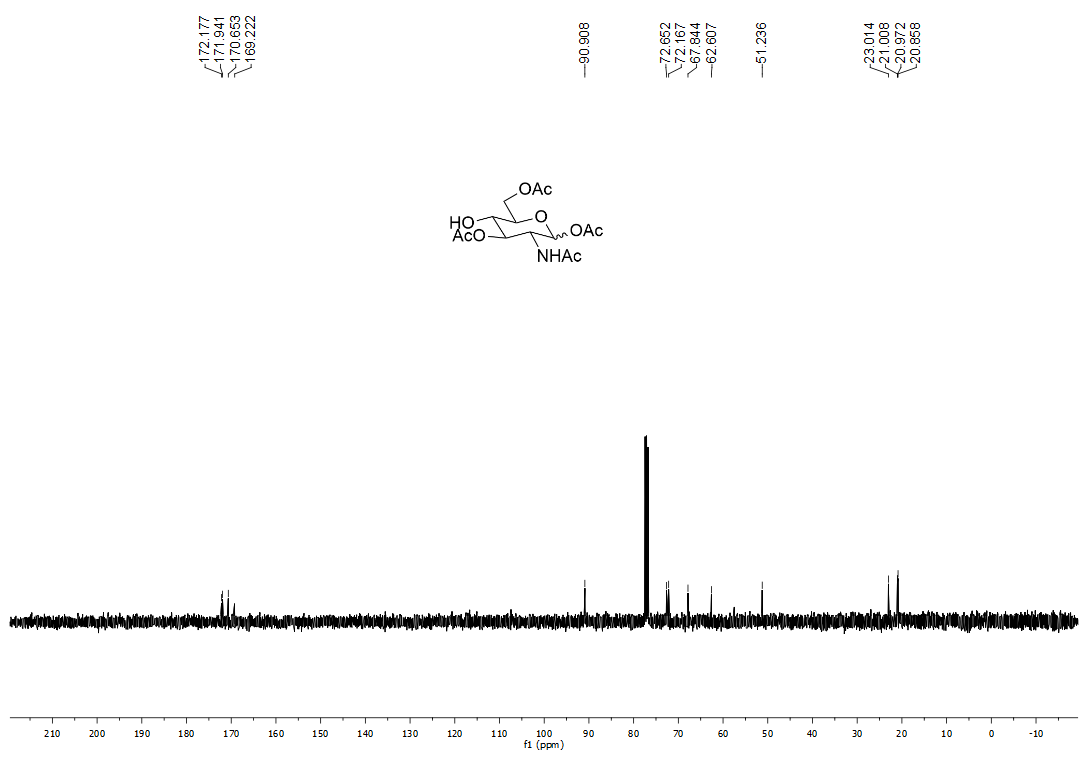


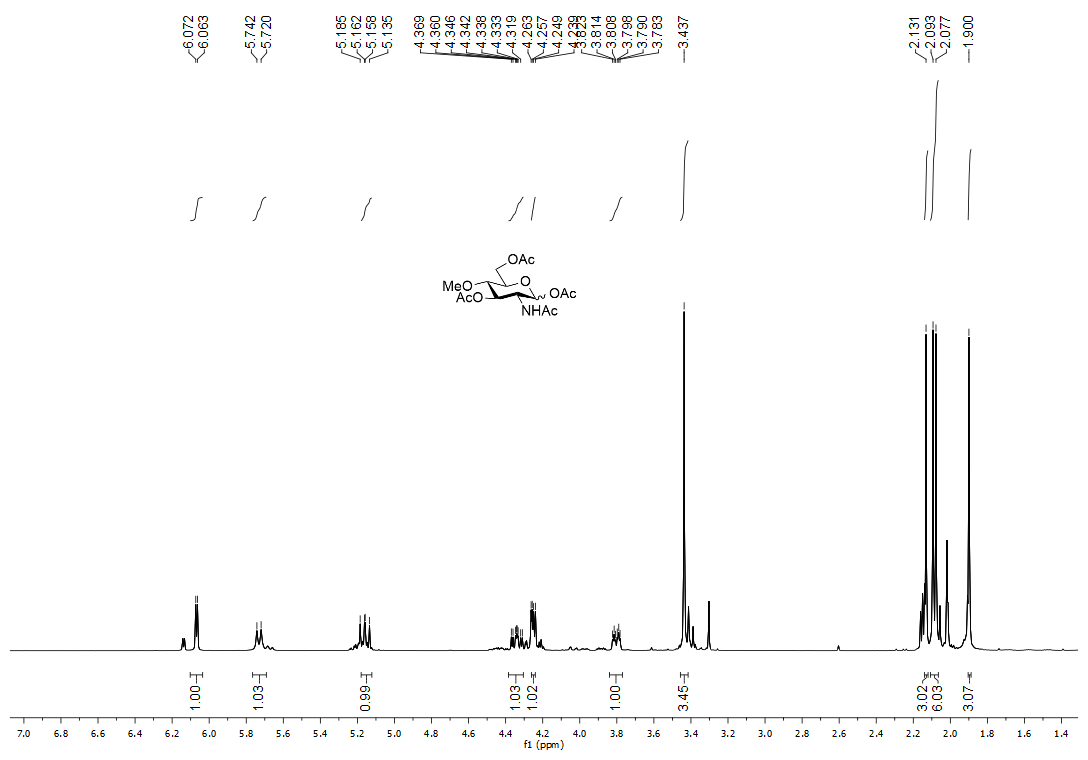


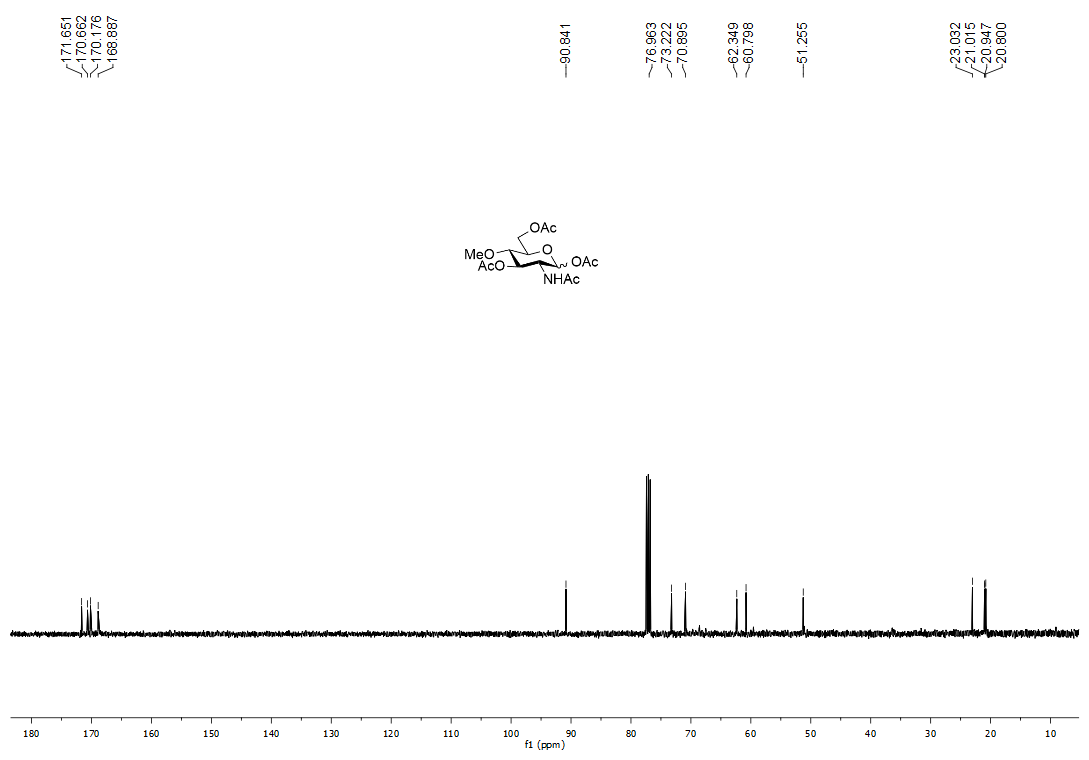


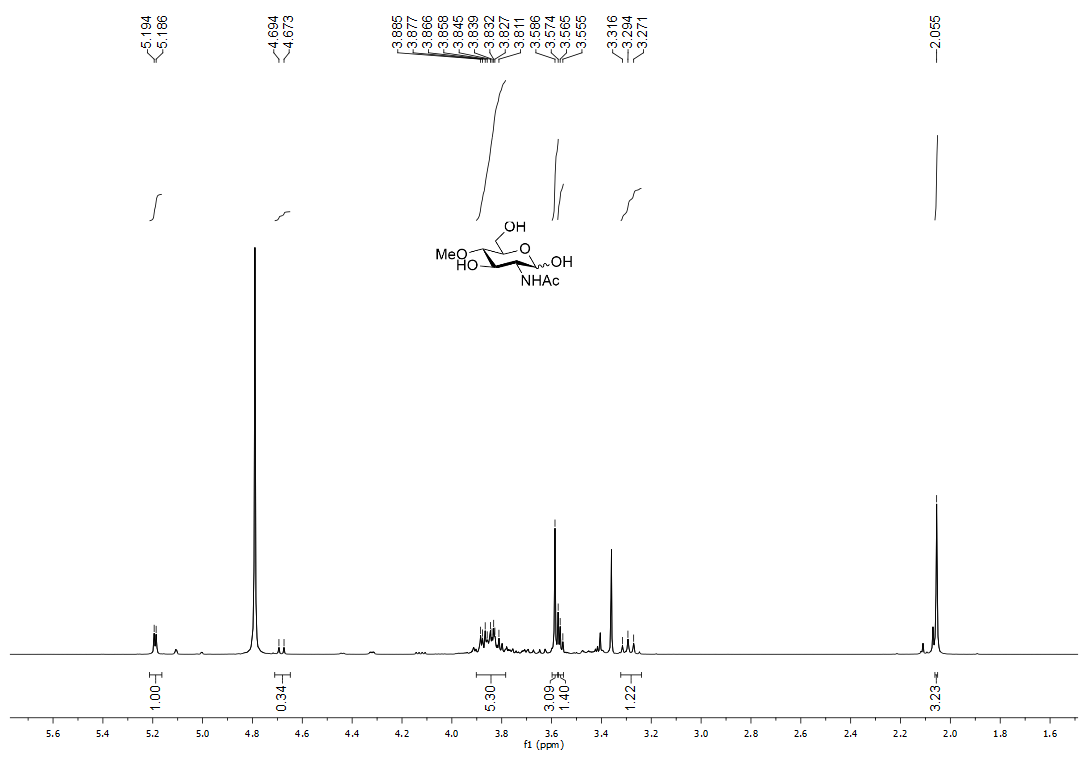


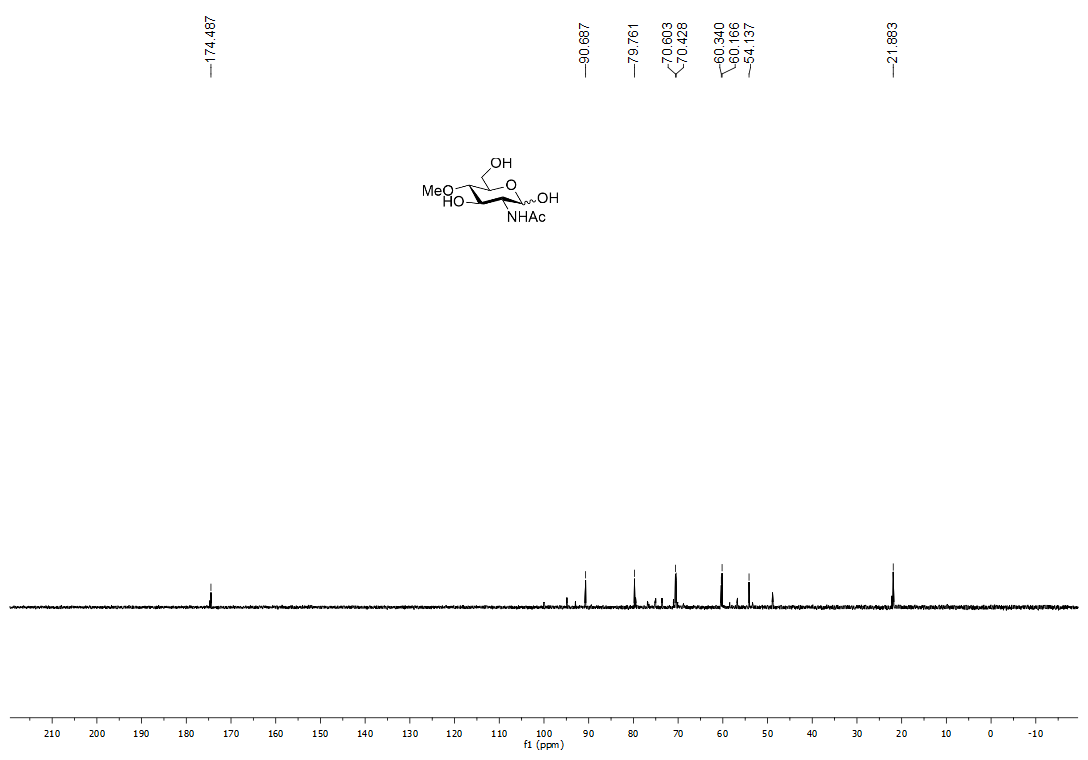

Supplement: Supplementary file 1 [file Table_1.docx]
